# Human-inspired time-series health evaluation with an adaptive multimodal electronic skin

**DOI:** 10.1126/sciadv.aeg5606

**Published:** 2026-07-31

**Authors:** Changhao Xu, Hongkai Zheng, Wenzheng Heng, Ruixiao Liu, John MarionSims, Jiahong Li, Peng Jin, Roland Yingjie Tay, Jihong Min, Guanzhi Wang, Yisong Yue, Wei Gao

**Affiliations:** ^1^ Andrew and Peggy Cherng Department of Medical Engineering, Division of Engineering and Applied Science, California Institute of Technology, Pasadena, CA, USA.; ^2^ Department of Computing + Mathematical Sciences, Division of Engineering and Applied Science, California Institute of Technology, Pasadena, CA, USA.

## Abstract

Electronic skin powered with artificial intelligence could enable next-generation robotic and medical devices, yet integrating multimodal sensors and analyzing heterogeneous, multifrequency time series remain challenging. Most wearable machine learning architectures are time-invariant and trained for a specific task, limiting transfer across modalities and users. We present a multimodal electronic skin that captures diverse physiological signs with an adaptive learning framework that rapidly generalizes to unseen tasks with minimal labeled data. Our streamlined end-to-end framework uses a spectral variational autoencoder to denoise and compress multifrequency biosignals into a shared, unified second-wise latent space that preserves the spectral-temporal structure, followed by a transformer to capture temporal dependencies to support diverse downstream tasks with data-efficient learning. We demonstrate robust adaptation with 94.7% accuracy in activity recognition and 90.2% precision in fatigue assessment across various users and daily activities regardless of device and user variations, highlighting a scalable route to generalized physiological time-series analytics and human performance assessments.

## Teaser

An adaptive electronic skin converts time-series wearable data into transferable latent codes for human performance evaluations.

## INTRODUCTION

Skin-interfaced wearable electronics hold immense potential in continuous health monitoring and personalized predictive analysis. These electronic devices, equipped with integrated analytical sensors, facilitate the convenient and noninvasive collection of comprehensive health profiles directly from the human body. Data collected from wearables have unlocked numerous applications with machine learning, including robotics (*1*, *2*), human-machine interfaces (*3*, *4*), disease prediction (*5*, *6*), and mental health evaluations (*7*).

Despite their transformative promise, three key challenges hinder the widespread adoption and real-world impact of such devices. First, most existing electronic skins are restricted to collecting few or single modality of data resulting from inherent hardware constraints (*8*, *9*). This limitation diminishes the richness of information, ultimately curbing the potential for comprehensive physiological monitoring. Second, the continuous data streams gathered by these devices are often fraught with noise and exhibit substantial variability in type, scale, frequency, and temporal dynamics. This heterogeneity poses complexities in downstream analysis, making traditional approaches such as a rule-based framework inadequate for processing the diverse and intricate data effectively. Existing machine learning techniques for biosensors, such as those based on regression or tree-based models, often process data points in isolation, failing to capture the inherent temporal correlations and dynamic nature of sequential health data (*9*). The cross-sectional approach undermines the potential of wearables to capture continuous physiological changes essential for evaluating human health dynamics (*10*). Furthermore, these conventional approaches often rely on handcrafted features extracted from the data, limiting their adaptability and scalability across the multifaceted demands of real-world applications. Representative wearable pipelines for activity recognition, fatigue assessment, and related human performance tasks typically rely on manual signal processing and task-specific machine learning models (table S1). Third, the process of generating and annotating sensor data is typically time-consuming and labor-intensive, restricting the availability of labeled datasets. Consequently, most existing methods are tailored for specific, narrow tasks, lacking the versatility needed to adapt to diverse real-world scenarios.

**Fig. 1.**
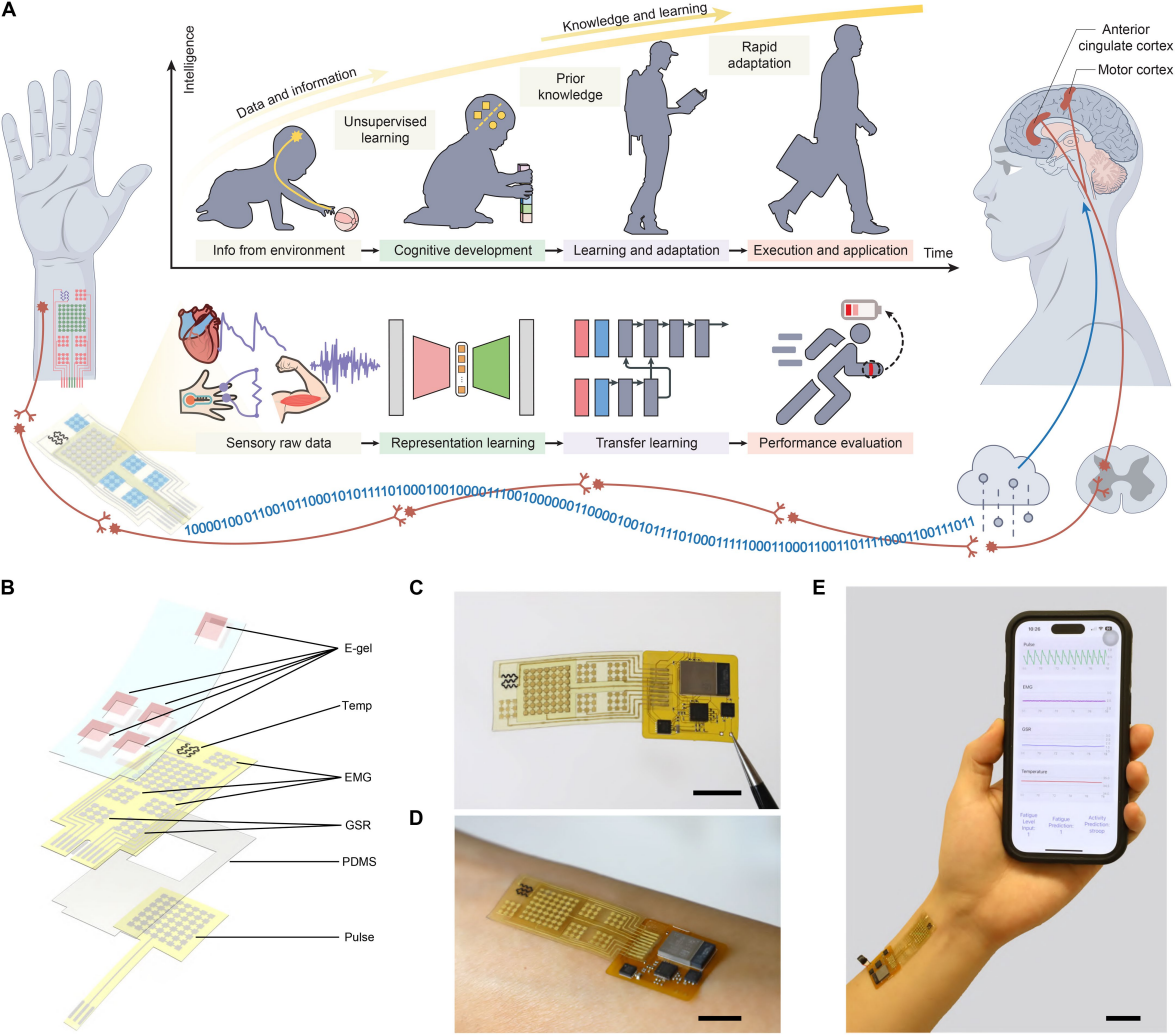
Illustration of human-inspired time-series health evaluation using a smart ARISE platform. (**A**) ARISE acquires knowledge through unsupervised learning and transfers it to previously unknown tasks with rapid adaptation, following a process analogy to human learning and generalization. (**B**) Exploded-view schematic illustration of the layered structure of the ARISE patch. (**C** and **D**) Optical images of the fully integrated wireless ARISE platform. Scale bars, 1 cm. (**E**) Photograph of the end-to-end process of wearable data collection, machine learning, cloud computing, data analysis, and real-time visualization on a mobile device. Scale bar, 2 cm.

Addressing these challenges necessitates the development of strategies that can replicate the way humans generalize in time-series data analysis (*11*). Humans naturally develop abstract concepts by interacting with their environment, learning from raw, unlabeled sensory inputs to form structured concepts that enable higher-level reasoning, classification, and prediction (*12*, *13*). Inspired by this human perceptual learning process, we aim to design an artificial intelligence (AI) framework for effective and versatile time-series health analysis and evaluation by first extracting unsupervised, abstract representations from unlabeled sensory data, which then serve as the foundation for downstream high-level predictive tasks such as activity recognition and fatigue assessment (Fig. 1A).

Recent breakthroughs in deep learning, especially in fields like natural language processing, demonstrate the potential of models trained on vast data to discern intricate patterns (*14*). These transformer-based models have shown proficiency as few-shot learners capable of predicting unseen data in generative AI contexts (*15*). The introduction of multihead attention within transformers has also reshaped the modeling of long-term dependencies in sequential data (*14*), making them highly effective for various downstream tasks, even when data are scarce (*15*). Although latent representation learning and transformer-based temporal modeling have been increasingly explored in general time-series analysis (*16*, *17*), their direct application to wearable physiological data remains challenging (*18*). Wearable biosignals differ fundamentally from conventional time-series benchmarks in that they lack standardized representations (*19*) and exhibit heterogeneous sampling frequencies, signal scales, noise structures, and physiological meanings across sensing modalities. Moreover, vast amounts of continuous time-series data in the wearable field are not readily available for training large models, unlike the extensive public text data in language models.

In this work, we present adaptive representation-learning integrated skin electronics (ARISE), a multimodal wearable platform that combines diverse physiological sensors with a streamlined machine learning framework for heterogeneous wearable time-series data recording and analytics. The ARISE system integrates sensors with varying modalities and sampling frequencies to create a holistic health profile.

The end-to-end human-inspired machine learning framework consists of two key components: task-agnostic representation learning, in which the model first learns general signal patterns from large amounts of unlabeled wearable data before any specific prediction task is defined, and supervised task-specific training. We first train a spectral variational autoencoder (SVAE), an unsupervised model that compresses noisy biosignal segments into compact latent representations while preserving their dominant temporal and spectral patterns. Unlike conventional windowing or generic embedding approaches applied to raw signals, the SVAE explicitly incorporates frequency-domain processing to capture quasiperiodic physiological dynamics and suppress noise and motion artifacts.

This design transforms multimodal biosignals into unified second-wise latent representations that are both compact and physiologically structured. These learned representations—internal summaries that retain the most informative patterns in the biosignals—are inherently robust to missing or corrupted data and enable rapid adaptation to unseen scenarios. Unlike conventional pipelines that rely on manually engineered features, our framework directly learns high-level physiological patterns from raw data, supporting scalable and flexible downstream analysis (*20*).

A key advantage of our approach is the creation of a shared latent space, that is, a common compact coordinate system in which signals from different sensors are organized according to their underlying physiological patterns while preserving meaningful temporal features and enabling generalization across various users and daily activities regardless of device and user variations. By leveraging transformer architectures, our model effectively captures complex temporal dependencies, ensuring accurate and rapid adaptation to task-specific inferences. In extensive validation studies spanning multiple daily tasks, ARISE achieved 94.7% accuracy in activity recognition and 90.2% precision in fatigue assessment, substantially outperforming conventional methods. Beyond these specific applications, our framework offers a scalable, generalizable approach for complex physiological time-series data analysis, with broad implications for personalized health care, human-computer interaction, and real-time biomedical monitoring.

## RESULTS

### Design and fabrication of the ARISE system

The ARISE consists of four sensor channels: peripheral pulse, galvanic skin response (GSR), electromyography (EMG), and skin temperature (Fig. 1, B and C). The core of the skin patch is built on a thin flexible polyimide substrate and mass fabricated at low cost through serial inkjet printing (fig. S1). The peripheral pulse sensor captures pulse waves above the radial artery. A polydimethylsiloxane (PDMS)–based airgap, spin coated and laser patterned, enhances dielectric sensitivity to 114.5% kPa^−1^ for a rapid and highly sensitive pressure response (fig. S2). The temperature sensor relies on inkjet-printed serpentine carbon as the sensing component and silver as interconnects, all encapsulated by polyimide and PDMS (fig. S3). EMG sensors monitor muscle engagement during wrist and hand movement, capturing signals from flexor digitorum profundus, flexor pollicis longus, and extensor digitorum communis, all of which can be measured by wrist EMG (*21*). To maximize EMG sensitivity while minimizing GSR interference, EMG electrodes were placed parallel to muscle fibers, whereas GSR electrodes are oriented perpendicularly (fig. S4).

Beyond traditional wired data transmission, the ARISE platform integrates wireless data transmission with a flexible printed circuit board for seamless data collection (Fig. 1, D and E). The electronics module consists of sensor readout circuitry, Bluetooth Low Energy (BLE) connectivity, and a compact cell battery for power supply. This lightweight flexible design enables conformal attachment to the human wrist, ensuring continuous, real-time physiological monitoring from human subjects in diverse settings. Compared with prior multimodal wearable platforms, ARISE is specifically designed as a reusable electronic skin for continuous human performance evaluation during daily wear (table S2).

### Representation learning of wearable time series

The ARISE continuously monitors four biosignals with varying sampling frequencies, modalities, and scales. This heterogeneity makes direct analysis challenging because of multimodal characteristics, variable temporal resolutions, and inherent noise. In addition, real-world sensory data often exhibit variability across individuals, environments, and different wearing locations. To address these challenges, we use unsupervised time-series representation learning, in which the model automatically learns compact summaries of the raw biosignals directly from data rather than relying on manually engineered features, to encode heterogeneous biosignals into a unified, second-wise representation. More specifically, we collected a large dataset of continuous multimodal biosignals from 50 healthy participants performing daily activities in both indoor and outdoor environments, exposing the pretrained model to substantial intersubject variability and diverse real-world conditions. These task-agnostic data collection and pretraining eliminate the need for manual annotation, making them highly efficient and scalable.

Present technologies often rely on extensive labeled data for each previously unknown user and task, restricting their broader adoption (*3*). Given the quasiperiodic nature of our collected biosignals like EMG and peripheral pulse, we introduce SVAE to efficiently encode the noisy time-series data into a low-dimensional latent space without manual feature engineering. The SVAE enhances the traditional variational autoencoder (VAE) architecture by incorporating frequency-domain processing to capture periodic and quasiperiodic temporal features of the signals more effectively. The SVAE applies a fast Fourier transform and frequency mode truncation to the encoder input, ensuring retention of only dominant frequency components while suppressing high-frequency noise. The decoder then applies an inverse fast Fourier transform to reconstruct the signal in the time domain.

**Fig. 2.**
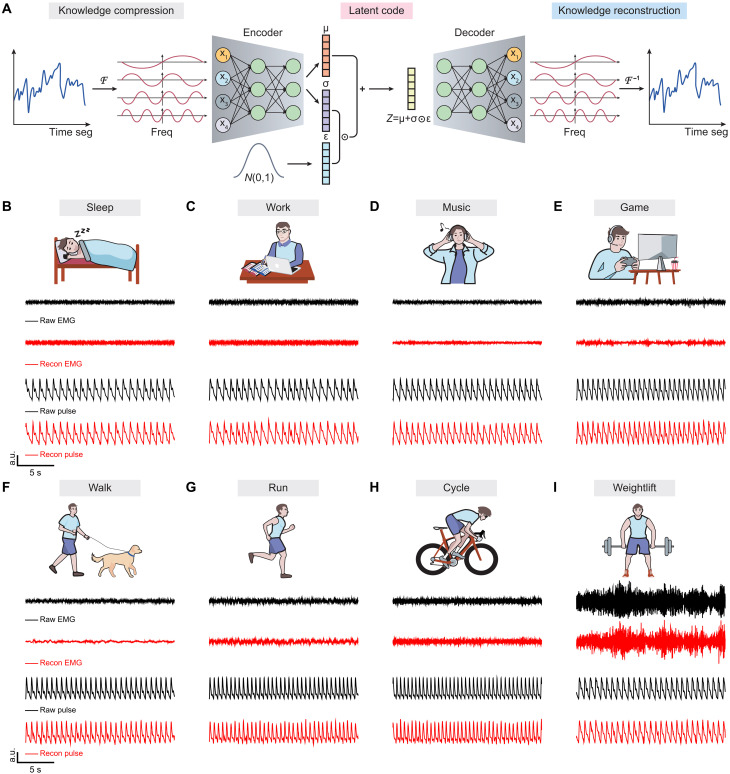
Representation learning of wearable time series. (**A**) Schematic of representation learning through knowledge compression and reconstruction in an SVAE architecture. (**B** to **I**) Comparison of raw and reconstructed peripheral pulse and EMG data during various daily activities, including sleep (B), work (C), listening to music (D), game (E), walk (F), run (G), cycle (H), and weightlift (I). The SVAE effectively reconstructed key temporal and spectral features of intricate wearable time-series data after knowledge extraction across multiple test scenarios. a.u., arbitrary units.

The training of SVAE follows the general principle of the VAE framework. The neural network–based encoder and decoder are trained to reconstruct input signals while regularizing the latent space to follow a known Gaussian structure (Fig. 2A and fig. S5) (*22*). The reconstruction loss consists of two components: reconstruction in the time domain, which ensures temporal fidelity, and reconstruction in the frequency domain, which preserves the key spectral features of the biosignals. Meanwhile, the regularization term is implemented as Kullback-Leibler (KL) divergence between Gaussians, which constrains the representation to a structured low-dimensional latent space. This not only fosters a shared latent space across different biosignals but also implicitly suppresses input noise, enhancing overall robustness and performance.

To assess its generalization capabilities, we evaluated SVAE on a diverse test set of unseen daily activities collected in both diverse indoor and outdoor scenarios. As shown in Fig. 2 (B to I), the SVAE accurately reconstructs the key temporal and spectral features of EMG and peripheral pulse data while effectively filtering out noise. The reconstructed signals retain their intrinsic temporal and spectral characteristics within typical frequency ranges, demonstrating the model’s robustness and zero-shot adaptation to unseen subjects and diverse real-world scenarios (fig. S6).

Once trained, the SVAE encoder transforms raw, noisy sensory data into compact representations over per-second intervals. This self-supervised representation learning serves as the foundation for downstream analysis tasks, as explored in later sections, enabling precise and data-efficient time-series analysis across multimodal biosignals.

### Long-term generalization and spectrogram analysis of wearable time series

The dynamic motion of hand movements presents substantial challenges in acquiring consistently stable raw data, leading to wearable datasets with missing data and motion artifacts. Spectrogram analysis plays a fundamental role in evaluating the robustness of signal reconstruction under such circumstances, particularly for validating the capture of characteristic frequencies that fluctuate over prolonged operations. To assess the long-term usability of the SVAE for continuous analysis, we analyzed spectrograms in the frequency domain for both raw and reconstructed data segments over extended periods.

**Fig. 3.**
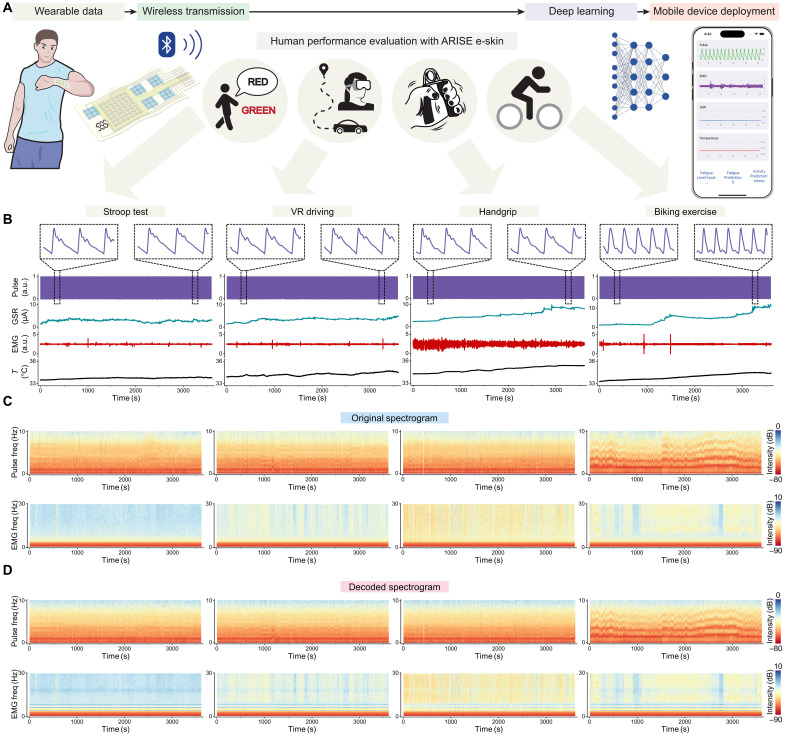
Long-term generalization and spectrogram analysis of wearable time series. (**A**) Schematic of the end-to-end development process of the ARISE system for time-series health evaluation. (**B**) Cross-activity on-body evaluation of the ARISE platform for human performance assessment, conducted through four controlled studies: Stroop test, VR driving, handgrip, and biking exercises. (**C** and **D**) Original (C) and decoded (D) spectrograms of the four controlled activities over a 1-hour testing period. The SVAE-generated continuous representation effectively reconstructs signals, preserving characteristic signals in the frequency domain while compressing excessive high-frequency details.

To facilitate real-time data visualization and user input, we developed a custom iOS app with a graphical user interface on mobile devices. Real-time collected health information is transmitted to a user interface through BLE, analyzed on a cloud infrastructure, and displayed on the mobile app (Fig. 3A and figs. S7 and S8). The seamless integration of a soft wearable device with real-time wireless communication enables continuous long-term health monitoring and insights evaluation (movie S1).

To further validate the system, we conducted multiple controlled human studies to demonstrate that the physiological information collected by the ARISE holds potential for a broad spectrum of personalized human performance monitoring applications, where subjects performed four distinct activities: the Stroop test, virtual reality (VR) driving simulation, handgripping, and biking exercise (movie S2). The Stroop test assessed cognitive function by requiring individuals to name the color of text that spells a different color. The VR driving simulation involved a racing game to emulate driver fatigue (*23*–*25*), with subjects using hand joysticks to control the vehicle. The handgrip and biking exercises, both physical tasks, required sustained exertion to induce fatigue (*26*, *27*). During physiological monitoring, the visual analog scale (VAS) was administered every 3 min to capture user fatigue perception with minimal interruption to ongoing task performance. Compared with longer multidimensional fatigue questionnaires, this low-burden format was more suitable for dense repeated labeling during the continuous 1-hour protocols used in this study (table S3). The VAS questionnaire was integrated into the mobile app for automated incorporation of user feedback with multimodal sensing. Note that the VAS scores were used as sparse anchor labels of perceived fatigue and linearly interpolated to generate second-wise regression targets. Thus, the model outputs represent continuous estimates of gradually evolving perceived fatigue constrained by intermittent self-reports, rather than independent second-by-second ground-truth measurements.

We recorded peripheral pulse, EMG, GSR, and skin temperature during all activities and plotted their variations over time (Fig. 3B). The periodic frequency components of pulse waveforms reflect the heartbeat, arterial wave reflections, and their harmonics. Given a typical heart rate of 50 to 150 beats per minute, the resulting fundamental frequency is relatively low. For EMG signals, the ARISE system’s uniquely low electrode-skin impedance substantially influences the energy spectrum, resulting in majority energy levels concentrated below 100 Hz (*28*). In addition, the EMG signal is primarily affected by the motor unit firing rate, which typically ranges from 0 to 20 Hz (*29*). We observed that for nonphysical activities such as Stroop tasks and VR driving, the basic spectrogram distributions of both pulse and EMG signals remained highly stable across subjects (figs. S9 and S10). During physical exercises like handgrip and biking, EMG variations reflected the activation of muscle motor units, while elevated pulse characteristic frequencies indicated increased heart rate and blood pressure.

The SVAE effectively learned the characteristic frequency patterns, encoding them as latent representations and then reconstructing them with minimal loss. As seen in Fig. 3 (C and D), the SVAE generated continuous representation that could reproduce signals accurately by preserving characteristic signals in the frequency domain while compressing the excessive high-frequency details. During the prolonged testing, the SVAE demonstrated exceptional robustness against missing data, motion artifacts, and sensor variability (figs. S11 to S14). To further examine practical sensing imperfections relevant to extended wear, we additionally evaluated representative artifact patterns including transient spikes, abrupt baseline changes, and baseline drift in both pulse and EMG signals (figs. S15 and S16). Even with fluctuating cardiac and muscle frequency components, the SVAE consistently captured the temporal dynamics of intricate multimodal data without observable signal drift, indicating its high efficiency and reliability in long-term physiological monitoring—achieved without the need for extensive manual data engineering.

### Rapid adaptation to activity recognition and human performance assessments

The self-learned compact representations from the SVAE effectively capture intricate temporal correlations and modality interactions present in large continuous wearable data and could serve as prior knowledge for a variety of downstream tasks. Given the transformer’s versatility and its ability to model complex dependencies, we use it as a general backbone, which leverages the unified and informative representations from the SVAE encoder to create a flexible, human-inspired framework that can adapt to diverse tasks with minimal training data (*14*).

To evaluate the effectiveness of these learned representations, we investigate activity recognition and fatigue assessment as two representative downstream tasks. These two tasks are highly relevant for behavioral monitoring and continuous human performance assessment. Activity recognition, which focuses on identifying the aforementioned activity classes in our experiments, forms the basis for behavioral analysis and health monitoring. In contrast, fatigue assessment involves quantifying the level of fatigue, defined as a psychophysiological condition characterized by reduced motor or cognitive performance (*30*). Fatigue manifests as decreased muscle activation from physical workload or mental exhaustion from cognitive overload (*31*), and it is a critical factor in settings demanding sustained physical or mental effort, such as sports, shift work, and military operations. Fatigue substantially affects various industries by reducing efficiency and increasing the risk of accidents. Critically, fatigue is implicated in about 20% of fatal road accidents (*32*), underscoring the need for effective monitoring and management systems to mitigate its deleterious effects on health and safety. Fatigue has been associated with biomarkers (*33*–*37*) and response time (*38*), yet most fatigue studies relied on single-parameter analyses or face recognition with computer vision, limiting their effectiveness (*39*).

**Fig. 4.**
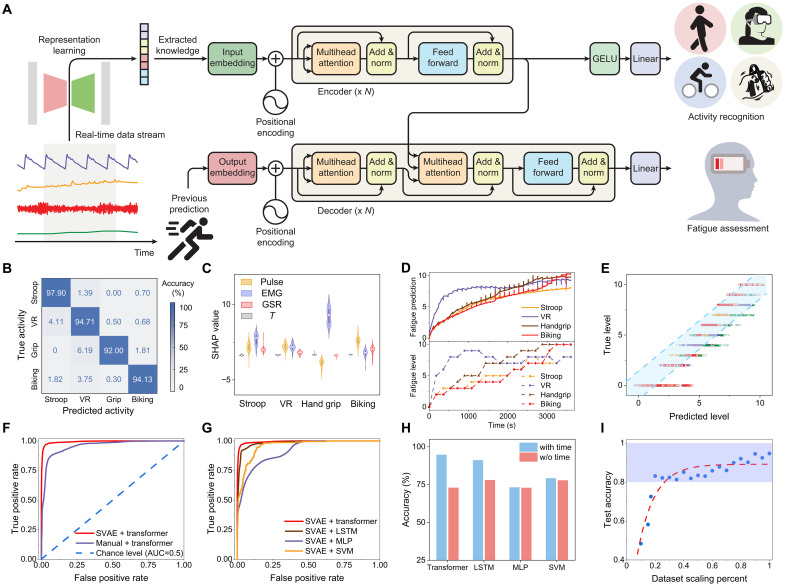
Systematic generalization and rapid adaptation of the SVAE-transformer model to downstream tasks. (**A**) Schematic of the end-to-end SVAE-transformer framework for human performance assessments. (**B**) Confusion matrix for activity recognition. (**C**) Shapley additive explanations analysis showing the relative contribution of multimodal sensors to different activities. (**D**) Psychophysiological fatigue level prediction using the transformer model. The model leveraged recognized activity to route task-specific predictions, estimating long-term fatigue levels from multimodal sensory data collected by ARISE. (**E**) Predicted versus true fatigue levels, demonstrating the model’s ability to predict fatigue over extended periods based solely on sensory data and prior predictions. (**F**) ROC curve comparing the SVAE-transformer model with transformers trained with handcrafted features, highlighting SVAE’s superior knowledge extraction over manually engineered features. AUC, area under the curve. (**G**) ROC curve comparison between the transformer and other machine learning models trained with SVAE-derived representations. LSTM, long short-term memory. (**H**) Comparison of model accuracy with and without temporal features across various machine learning methods. (**I**) Model accuracy at varying proportions of training data.

In the activity recognition experiment, the objective is to predict the activity on the basis of the time-series data collected by the ARISE. The pretrained SVAE first processes the multimodal sensory data stream, generating unified representations across different sensors. These representations are then concatenated into a single token, forming a compact and information-rich input for classification. The classification task is performed using a transformer-based model consisting of a four-layer transformer encoder backbone followed by a linear classification head (Fig. 4A and fig. S17). The model processes 120 tokens (equivalent to 2 min of data) and outputs class probabilities. This second-wise tokenization provides a shared temporal grid for multimodal biosignals with different sampling frequencies while keeping the transformer input compact. Training is conducted using the standard cross-entropy loss with mixup regularization (*40*). As illustrated in Fig. 4B, our transformer-based model achieves 94.7% test accuracy across four different daily activity classes. To further investigate sensor contributions, we use additional analysis using Shapley additive explanations. As shown in Fig. 4C, EMG and peripheral pulse sensors have the highest impact, while the temperature sensor contributes minimally. This observation aligns with physiological expectations: EMG and pulse signals vary substantially across activities, whereas the skin temperature remains relatively stable (figs. S18 to S20).

The transformer model further quantifies fatigue levels by leveraging the activity recognition predictions to guide task-specific physical and mental fatigue assessment. The task protocols were designed to maintain relatively controlled demands within each activity such that fatigue accumulated progressively over sustained task performance. Accordingly, the model was intended to capture fatigue-related physiological changes within activity-specific contexts rather than relying on task duration alone. In the fatigue assessment task, the objective is to predict the long-term fatigue level of a subject on the basis of the continuous stream of multimodal sensory data collected by ARISE. The SVAE encoder again generates unified representations, which are concatenated to form continuous tokens for each time point. The model for fatigue assessment uses a standard transformer with an encoder-decoder structure. The encoder processes 120 continuous tokens (equivalent to 2 min of data), while the decoder incorporates the previous eight prediction tokens to capture long temporal dependencies over time. The model is trained using the mean squared error loss with C-mixup regularization (*41*). During inference, the model predicts fatigue levels of the subject over a 1-hour window, a particularly challenging task because of the need for long-term rolling predictions based solely on continuous sensory data, distinct from the classic time-series forecast or imputation problems (*42*, *43*), where the model can directly access look-back data of the variable we need to predict. Instead, the model predicts fatigue progression using only sensory data and previous predictions. As demonstrated in Fig. 4 (D and E) and fig. S21, our models can achieve an average 90.2% accuracy across various activities. These results highlight ARISE’s ability to transform continuous physiological signals to meaningful health insights, enabling potential for robust human performance assessments in real-world settings.

### Ablation studies

Ablation studies are conducted to gain deeper insights into our methods and assess the contribution of key components in our end-to-end framework.

#### Effectiveness of SVAE representations

The pretrained SVAE uncovers complex underlying patterns in sensory data—avoiding the limitations of handcrafted feature engineering that focuses only on predefined characteristics based on domain knowledge, making it ideal for fully exploiting the rich and diverse information embedded in sensory data and quickly adapting to previously unknown tasks with few-shot trials. To assess the advantage of representation learning, the first question we investigate is how the learned representations from the SVAE encoder compare to handcrafted features derived from traditional data engineering techniques. For this experiment, we use feature engineering strategies from the literature to encode EMG and peripheral pulse data into second-wise representations, extracting meaningful summary features such as period, peak values, and statistical metrics (*7*, *44*). We then perform a controlled experiment on the activity recognition task, training the same transformer-based model with these handcrafted features and comparing it to the model trained with SVAE representations. As shown in Fig. 4F, models trained with SVAE representations substantially outperform those relying on handcrafted features, both in terms of overall accuracy and receiver operating characteristic (ROC) curves. This observation is consistent with prior wearable studies, many of which rely on manually engineered statistical, spectral, or physiological features for downstream prediction (table S1). These results demonstrate that our approach not only eliminates the labor-intensive process of feature engineering but also yields superior representations for downstream tasks.

#### Importance of modeling temporal dependencies with transformers

Another question we examine is the importance of modeling temporal dependencies using transformer architecture in sensory data. To assess this with controlled experiments, we first modify the input to the activity recognition model to include only a single token per prediction, effectively removing access to temporal correlations across different time steps. We compare the performance of multiple models with this constraint to those trained with access to multiple tokens as context. For this study, we first consider multiple models, including our transformer-based model and traditional time-invariant methods such as multiclass support vector machines (SVMs) and multilayer perceptrons (MLPs), as well as sequential models such as recurrent neural networks (RNNs). Notably, the transformer-based model outperforms the traditional methods by a large margin (Fig. 4G). While RNNs capture temporal dependencies, they struggle to explicitly model relationships between variables in multivariate time series (*18*). Therefore, attention-based networks also showed much stronger systematicity than neural networks trained in standard ways. To further contextualize the performance against recent time-series architectures, we additionally compared the transformer with PatchTST (*45*), TimesNet (*17*), and iTransformer (*46*) under the same SVAE-derived representations (fig. S22). The lightweight transformer achieved comparable or superior ROC performance relative to these recent models, further supporting the effectiveness of combining compact physiological representation learning with temporal attention-based modeling.

We further compare our transformer model with and without temporal information. As shown in Fig. 4H and fig. S23, all temporal models experience performance degradation when deprived of temporal dependency modeling, whereas classical models remain unaffected because of their inherent lack of time-aware mechanisms. These results highlight the critical role of temporal dependencies in sensory data analysis and demonstrate the transformer’s powerful temporal modeling capability through its attention mechanism.

#### Sensitivity to temporal resolution

We further evaluated whether the downstream model was sensitive to the temporal resolution of the SVAE-derived tokens. Starting from the baseline second-wise latent representations, we temporally pooled consecutive tokens to form 2-, 5-, and 10-s representations while keeping the physical input window fixed at 2 min. This resulted in 120, 60, 24, and 12 tokens per input window, respectively. As shown in fig. S24, the model maintained comparable performance at 1- and 2-s resolutions, whereas coarser resolutions led to reduced performance. These results indicate that second-wise tokenization is an effective design choice, as it preserves short-term physiological dynamics while maintaining compact transformer inputs for temporal modeling.

#### Data efficiency

Last, we investigate the data efficiency of our SVAE-transformer hybrid model. While transformer-based approaches typically require vast amounts of data, SVAE reduces computational overhead by compactly encoding time-series data before feeding it into the transformer. To evaluate this, we train the model on subsets of the training data with varying sizes and measure its accuracy. As shown in Fig. 4I, the model demonstrates strong performance even in few-shot data regimes, achieving ∼80% accuracy with only 30% of the full training data. As the size of the training set increases, the model gradually improves and reaches its best performance with the full dataset. These results demonstrate the data efficiency of our SVAE-transformer based approach, affirming its ability to generalize with limited labeled data while continuing to improve with larger datasets.

## DISCUSSION

This work demonstrates a monolithic wearable platform that is capable of human performance predictions through an end-to-end machine learning framework. The ARISE platform is user-friendly for daily monitoring, featuring wireless data transmission and real-time visualization on a mobile app. Moreover, it can be fabricated at low cost and scale through inkjet printing. ARISE uses a fully integrated multimodal electronic skin for physiological signal acquisition, a representation learning architecture for translating high-frequency physiological signals into low-dimensional compact knowledge, and a time-series transformer–based deep learning model for evaluating human performance.

We designed the system to fully leverage easily accessible unlabeled wearable data, showing that unsupervised deep learning can integrate and extract time-series wearable data. The SVAE model demonstrated robust learning of signal patterns by preserving characteristic frequencies while removing excessive details. Unlike conventional machine learning approaches, our strategy models the temporal dependencies inherent in time-series data and minimizes human labor in manual labeling and data engineering. With the prior-learned SVAE network enabling meta learning, a subsequent transformer model can rapidly learn to evaluate unknown time-series tasks with minimal training data. This platform achieves more than 90% accuracy in human performance assessments, including activity recognition and fatigue level prediction. Combining SVAE with time-series transformers represents a major step in data-efficient AI for wearables that understands and predicts human physiological patterns over time, akin to how humans integrate and generalize information. Compared with conventional signal processing methods, our framework shifts the workflow from manually engineered features toward task-agnostic representation learning directly from unlabeled multimodal time-series data, followed by transformer-based temporal modeling. This design reduces dependence on labor-intensive feature engineering, improves flexibility across downstream tasks, and better accommodates heterogeneous and temporally evolving wearable signals. At the same time, the present approach remains less directly interpretable than explicit engineered features and requires sufficient pretraining data and computational resources. From a deployment perspective, the soft, lightweight, and wireless ARISE platform is promising for continuous daily use. Our long-term experiments and artifact analyses indicate robustness to common signal perturbations, although future studies should further evaluate long-term wearability, sensor stability, and model performance across broader populations, additional cognitive and physical tasks, and unconstrained real-world conditions. In addition, because fatigue labels were based on repeated VAS self-reports, they reflect perceived fatigue and may vary across individuals. This low-burden format enabled dense sampling with minimal task disruption. Future studies could incorporate objective fatigue markers and larger cohorts. Our end-to-end SVAE-transformer framework is well suited for scalable, generalized human performance assessments, providing valuable insights into the complex dynamics of human physiology and enabling informed decision-making.

## MATERIALS AND METHODS

### Fabrication of the electronic skin

The fabrication process for the electronic skin started on a silicon wafer with 300-nm silicon dioxide (University Wafer), which was used as a substrate without any further treatments. First, polyimide solution (PI-2611, HD Microsystems) was spin coated on the silicon wafer with a 4-μm thickness and annealed at 300°C for 30 min. The sensing electrodes of both top and bottom layers were fabricated via serial printing of silver and carbon using an inkjet printer (DMP-2850, Fujifilm). The inkjet-printed patch was annealed at 250°C for 30 min. Laser cutting (Universal Laser System) was then performed to obtain the desired electrode patterns. A PDMS (Sylgard 184, Dow Corning, 12:1) thin film was spin coated (600 revolutions per minute, 30 s) onto both top and bottom layers and half cured at 60°C for 20 min. Serial laser cutting was subsequently performed to first cut the PDMS openings and then cut the electrode outlines. The top layer of the electronic skin was then picked up carefully with a glass plate and dry transferred onto the bottom layer. The transferred patch was then fully cured at 70°C for 1 hour. A final PDMS encapsulation layer was spin coated and patterned on the backing of the assembled electronic skin to prevent airgap leakage. Silicone adhesives (Dupont) and electrolyte gel (SignaGel, Parker Laboratories Inc.) were cast onto the patch before the patch was placed onto human subjects.

### Electronic system design and integration

The circuit architecture comprises three core functional blocks: power management and sensor interfaces, data processing and wireless communication, and functional readout modules. The power management unit uses a voltage regulator (LD39050PU33R, STMicroelectronics) to stabilize the battery output at a constant 3.3 V. At the heart of the system lies a compact wireless module (STM32WB5MMG, STMicroelectronics) that integrates a 32-bit Arm Cortex-M4 microcontroller unit with BLE 5.0 radio, handling both data processing and wireless communication. This module is programmed through an ST-LINK/V2 in-circuit debugger (STMicroelectronics). For sensor interfacing, the microcontroller unit measures the resistance of temperature and GSR sensors using a voltage divider circuit and its built-in 12-bit analog-to-digital converter. Pulse sensing is implemented with an FDC1004 capacitance-to-digital converter chip (FDC1004, Texas Instruments) using I2C communication for data acquisition. EMG signals are captured using an AD8232 bioelectric readout chip (AD8232, Analog Devices), with signal conditioning parameters optimized before analog-to-digital conversion. The integrated multimodal dataset is wirelessly transmitted via BLE to a mobile device, where custom-developed software algorithms perform further calibration and analysis. This architecture ensures reliable data acquisition and processing across multiple physiological parameters.

### Sensor characterization

Sensor characterizations, including the capacitance of the peripheral pulse sensor, as well as the current and voltage of all other sensors, were measured by the data acquisition and multimeter system (4200-SCS, Keithley) and the wireless flexible printed circuit board system. Temperature sensor characterization was performed on a ceramic hot plate (Thermo Fisher Scientific).

### Human studies and data collection

The human studies of electronic skin for human performance monitoring were in compliance with the protocols (24-0892 and 19-0895) approved by the Institutional Review Board at the California Institute of Technology (Caltech). In total, 50 healthy subjects were involved in the human studies and data collection. The participants (age range of 20 to 36 years) were recruited from the Caltech campus and the neighboring communities through advertisement by posted notices, word of mouth, and email distribution. All participants gave written informed consent before participation in the study.

### Daily activities with an unlabeled dataset

During the study, participants wore the electronic skin and performed daily routine activities, including reading, watching movies, working, or gym exercises such as weightlifting and running. Each subject participated in either lab or gym wearing the system for around 1 hour. The pulse and EMG data were collected in real time. To ensure data quality over the entire testing period, sensor data were collected to a laptop through wired connections.

### Controlled studies

Stroop test is a widely used psychological test in clinical practice, in which the subjects are required to name the color of the word when the color does not match the name of the color. The test was taken in a custom-developed computer game, where the successful response reduces the time allowed for the next round, and failed response extends the time allowed to respond. Such dynamic adjustments ensure an intense and steady gaming experience across subjects with varying reaction speed and record the fatigue level periodically.

For the VR studies, we applied an Oculus Quest 2 VR headset as the testing device. The subjects were asked to play a virtual driving test (Mini Motor Racing X) throughout the 1-hour period. The subjects could use their thumbs to control the steering and acceleration of their vehicles during the game. A handgripper with adjustable resistance was used for the handgrip test, where the subjects were asked to compress and hold the handgrip steady for as long as possible during the 1-hour test period. For the biking exercise, the subjects were asked to perform a constant workload cycle ergometry at a self-selected pace.

Each activity was conducted for 1 hour, and participants were given sufficient rest before each activity to ensure that the physiological state from a previous state did not affect the subsequent activity. During each activity, the biosignals from our electronic skin ARISE were continuously recorded. Participants self-reported their fatigue levels on a scale from 1 to 10 every 3 min. For model training and evaluation, these discrete VAS scores were used as anchor points and linearly interpolated to generate second-wise fatigue labels. This interpolation assumes that perceived fatigue changes gradually over the sustained task protocol, which is consistent with the controlled experimental design. The resulting second-wise labels were aligned with the second-wise multimodal representations generated by the SVAE encoder. A standard deviation of 1.4 from the prior VAS-based fatigue literature was adapted to quantify the prediction accuracy (*47*, *48*).

### Data processing

During the on-body trial, the collected multimodal data were transmitted through either wires or wireless Bluetooth communications in real time, followed by data extraction and calibration. To reduce motion artifacts and reproducibility concerns of human subject variations, all raw pulse waveforms were filtered and normalized through a custom-developed iterative baseline correction algorithm. For the EMG data, we further filtered out the 60-Hz powerline interference. To give a direct comparison, the data processing pipeline consists of both normalized raw data and previously state-of-the-art handcrafted feature extraction. Data were processed using Microsoft Excel and Origin. The end-to-end learning framework was trained on Nvidia A100 GPU.

### Representation learning via SVAE

The SVAE is extended from the traditional VAE framework and is designed to effectively encode heterogeneous, multimodal sensory data into a compact and robust unified representation. It consists of three components: encoder, latent space sampling, and decoder.

Given a univariate time series $=\left\{x_{1},x_{2},\ldots ,x_{T}\right\}$, the encoder outputs the distribution of the latent space, which is a multivariate Gaussian with a diagonal covariance structure. Specifically, it first transforms x into a sequence of complex numbers $\left\{X_{1},X_{2},\ldots ,X_{T}\right\}$ in the frequency domain using the fast Fourier transform. We then define a cutoff frequency index K and discard all frequency components above K, which results in a sequence with 2K elements $X=\left\{X_{1},X_{2},\ldots ,X_{2K}\right\}$. This sequence is then mapped into a mean vector $\mu \in R^{d}$ and a log variance vector $\nu \in R^{d}$ through a neural network, as illustrated in fig. S5. The latent space sampling samples the latent code from the output distribution as z=μ+ν⊙ϵ, where ϵ∼𝒩(0,I). Given the latent code, the decoder first produces $\hat{X}=\left\{\hat{X_{1}},\hat{X_{2}},\ldots ,\hat{X_{2K}}\right\}$ as the reconstruction of the frequency-domain representation of the input through a neural network symmetric to that of the encoder. An inverse fast Fourier transform is then applied to convert the reconstructed representation $\hat{X}$ back to the time domain $\hat{x}=\left\{\hat{x_{1}},\hat{x_{2}},\ldots ,\hat{x_{T}}\right\}$.

The training objective of SVAE consists of three components: time-domain reconstruction loss, frequency-domain reconstruction loss, and KL divergence regularization. The overall objective is given by

$$L\left(x\right)={|x-\hat{x}|}_{2}^{2}+\lambda_{1}{|X-\hat{X}|}_{2}+\lambda_{2}L_{\text{KL}}$$

where $L_{\text{KL}}=-\frac{1}{2d}\sum_{i=1}^{d}\left[1+\nu_{i}-\mu_{i}^{2}-\text{exp}\left(\nu_{i}\right)\right]$ and $\lambda_{1},\lambda_{2}$ are hyperparameters. The model is trained on a large corpus of unlabeled sensory data collected from diverse activities and subjects detailed above using the Adam optimizer (*49*).

Once trained, the encoder, combined with the latent space sampling method described above, is used to encode the raw signal x into its latent representation z. In this work, we process the continuous data stream by dividing it into second-wise segments, with each segment of the raw signal being encoded into a compact, second-wise latent representation. This approach enables consistent and efficient representation of time-series data for downstream analysis.

In this work, we train SVAEs for EMG and peripheral pulse signals as their sampling frequencies are much higher than 1 Hz. For EMG, we set the cutoff frequency index K=250, the dimensionality of the latent code d=64, and loss weights $\lambda_{1}=0.01$ and $\lambda_{2}=0.005$. For peripheral pulse, we set K=32, d=16, $\lambda_{1}=0.01$, and $\lambda_{2}=0.1$.

### Activity recognition via encoder-only transformers

For the activity recognition experiments, we use encoder-only transformer architecture to perform the classification task.

#### Input representation

The raw multimodal sensory data are first processed through the SVAEs to obtain unified, second-wise representations. For each second, the representations from four sensor modalities are concatenated into a single continuous token, capturing the combined physiological signals at that second. We add the standard positional encoding to each token, which is a common technique to indicate the position of each token in the input sequence. In addition, we also encode the timestamp information through learnable linear embedding layers and add to each token to inform the relative time step of each token from the starting point.

#### Neural network architecture

The backbone of the network is a stack of standard transformer encoder blocks, as illustrated in fig. S14. A linear classification head is appended to map the encoded representation into activity class probabilities. We use four blocks in our experiments, set the embedding dimension to 512, and use eight attention heads.

#### Training process

The model is trained on a relatively small, labeled dataset detailed above. The context length is 120 (equivalent to 2 min of contextual data) to ensure sufficient temporal context for accurate classification. The training objective is the standard cross-entropy loss. To improve generalization and prevent overfitting, mixup regularization is applied during training (*40*). This technique involves randomly interpolating between pairs of examples and their labels, encouraging the model to learn more robust features. The model is trained using the Adam optimizer with an annealed learning rate schedule.

#### Inference process

During inference, the trained transformer encoder can process incoming second-wise tokens in real time or batch mode. For each 2-min window of data, the model outputs the predicted activity class on the basis of the learned temporal patterns.

### Fatigue assessment via encoder-decoder transformers

For the fatigue assessment experiment, we adopt the standard encoder-decoder transformer. This structure allows the model to capture complex temporal dependencies and trends in the data, making it well suited for continuous fatigue quantification. We use the same input representation described in the previous section, where raw sensory data are transformed into unified, second-wise representations using the pretrained SVAEs.

#### Neural network architecture

The backbone of the network consists of a stack of transformer encoder blocks followed by a stack of decoder blocks, as illustrated in fig. S14. The encoder again processes sequences of 120 tokens into encoded features. The decoder takes the encoded features and previous fatigue level predictions as input, allowing the model to incorporate historical prediction trends. The look-back window for fatigue predictions is set to 8 s, meaning that the model considers the last 8 s of predicted fatigue levels when making subsequent predictions. The output of the decoder is then fed into a linear projection head, which predicts the continuous fatigue level for each second.

#### Training process

The model is trained to predict fatigue levels over time using the collected dataset with labels detailed in the previous section. The model is optimized using mean squared error loss, which penalizes deviations between predicted and actual fatigue levels. We use C-mixup regularization to enhance generalization (*41*). This method is a counterpart of the original mixup regularization for regression tasks, which randomly interpolates between similar samples in both input and label space. The Adam optimizer with an annealed learning rate schedule is again used to optimize the model.

#### Inference process

During inference, the trained encoder-decoder transformer uses the most recent 2-min window of the sensory data along with the last 8 s of predicted fatigue levels to predict the fatigue levels. For each second within the 2-min window, the model predicts the corresponding fatigue level.

## Supplementary Materials

### The PDF file includes:

Figs. S1 to S24 Tables S1 to S3 Legends for movies S1 and S2 References
