# Supplementary material for: Human-inspired time-series health evaluation with an adaptive multimodal electronic skin
Source: Sci Adv. 2026 Jul 31;12(31):eaeg5606. doi: 10.1126/sciadv.aeg5606 (PMC13426441; doi:10.1126/sciadv.aeg5606)
Supplement: Supplementary file 1 — Figs. S1 to S24 Tables S1 to S3 Legends for movies S1 and S2 References [file sciadv.aeg5606_sm.pdf]

Science Advances
AAAS

Supplementary Materials for

Human-inspired time-series health evaluation with an adaptive multimodal electronic skin

Changhao Xu *et al.*

Corresponding author: Wei Gao, weigao@caltech.edu

Sci. Adv. **12**, eaeg5606 (2026)
DOI: 10.1126/sciadv.aeg5606

The PDF file includes:

Figs. S1 to S24
Tables S1 to S3
Legends for movies S1 and S2
References

Other Supplementary Material for this manuscript includes the following:

Movies S1 and S2

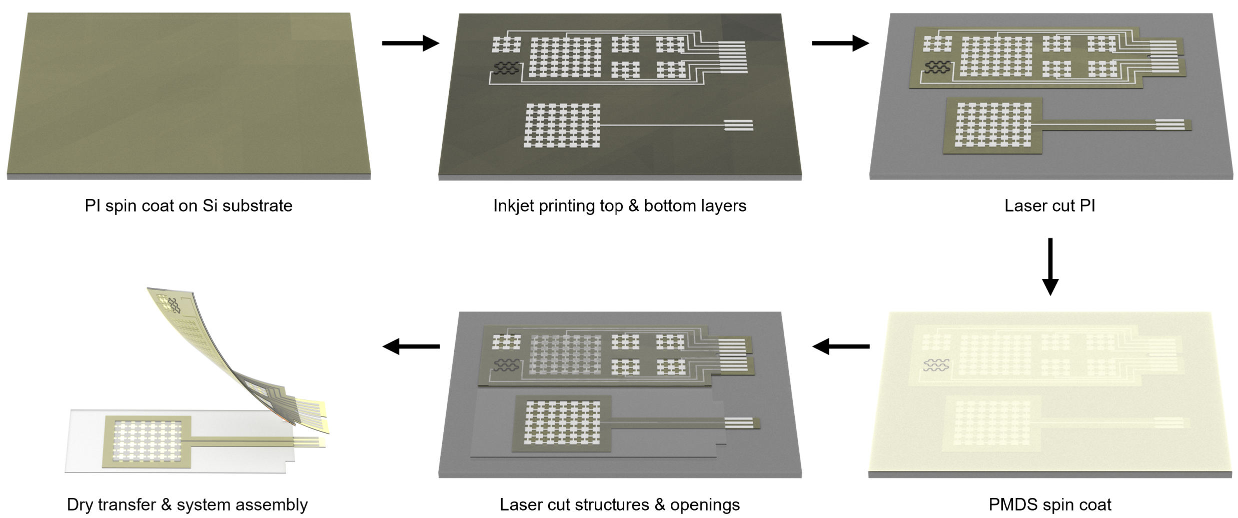

Fig. S1. Fabrication process of the inkjet-printed flexible ARISE patch.

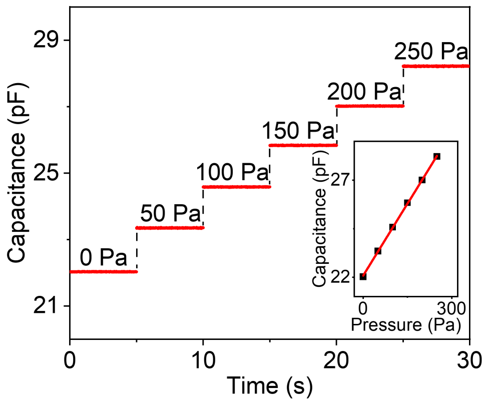

Fig. S2. Characterization of peripheral pulse sensor.

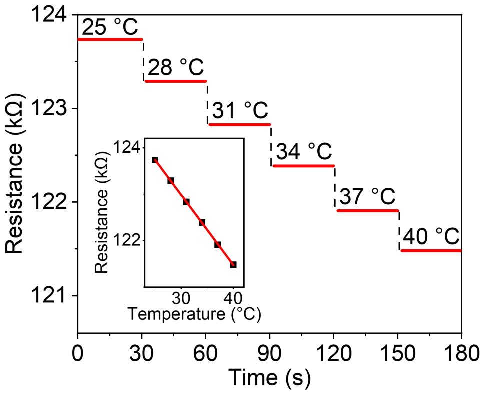

Fig. S3. Characterization of skin temperature sensor.

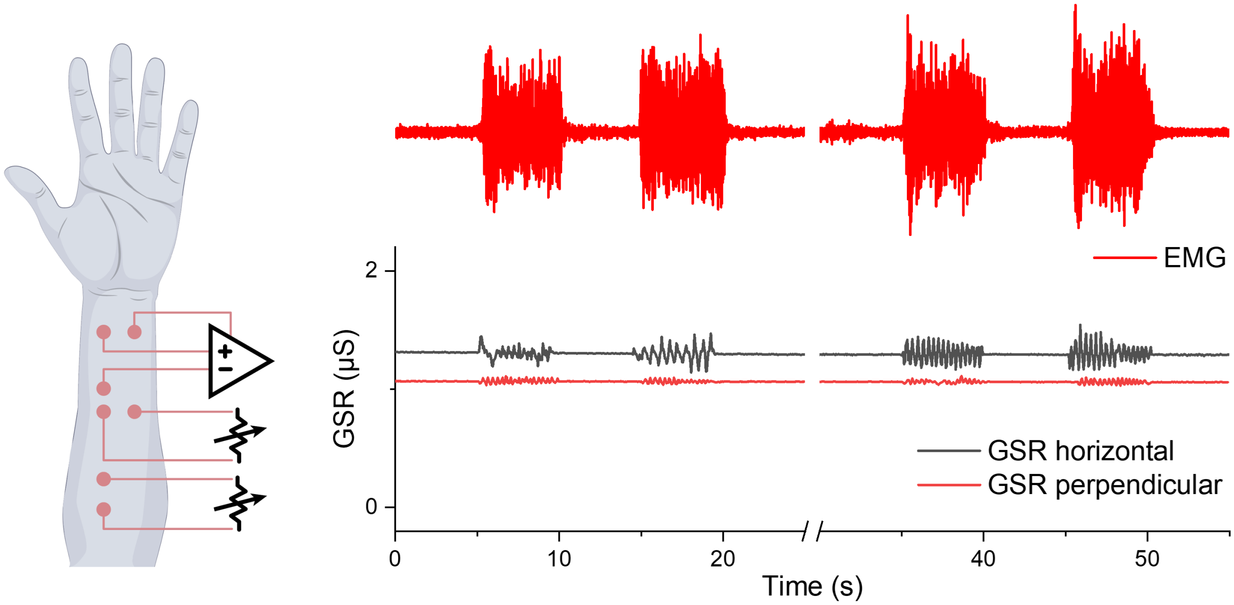

Fig. S4. Characterization of GSR independence with EMG. When placed perpendicular to muscle fibers, GSR defects are minimized against muscle actions.

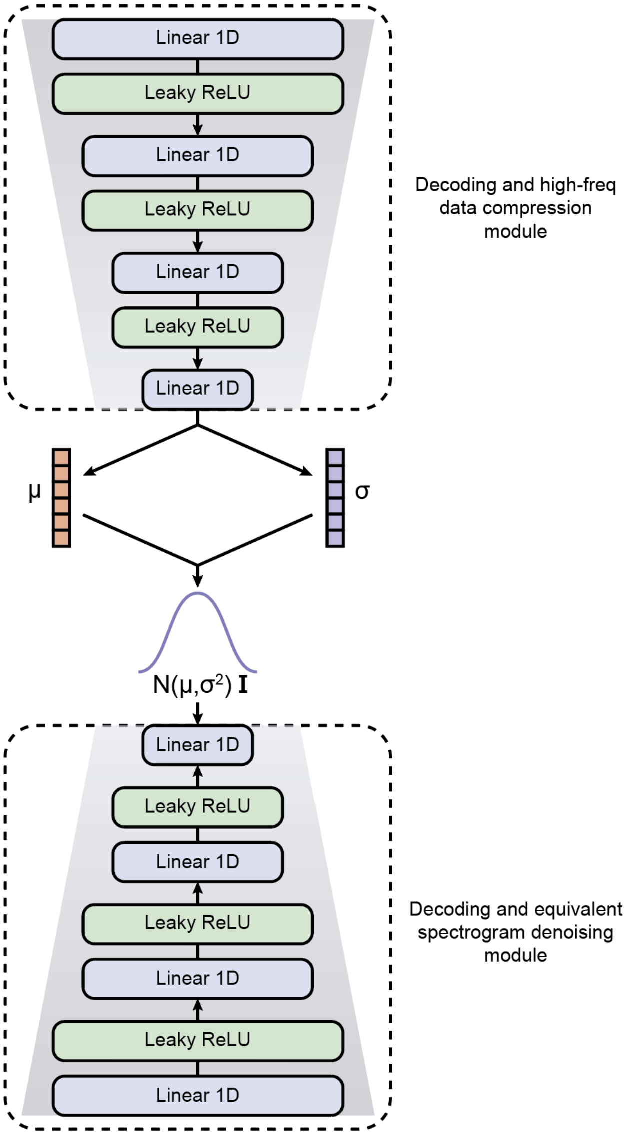

Fig. S5. Details of the variational autoencoder (VAE) architecture in SVAE for representation learning.

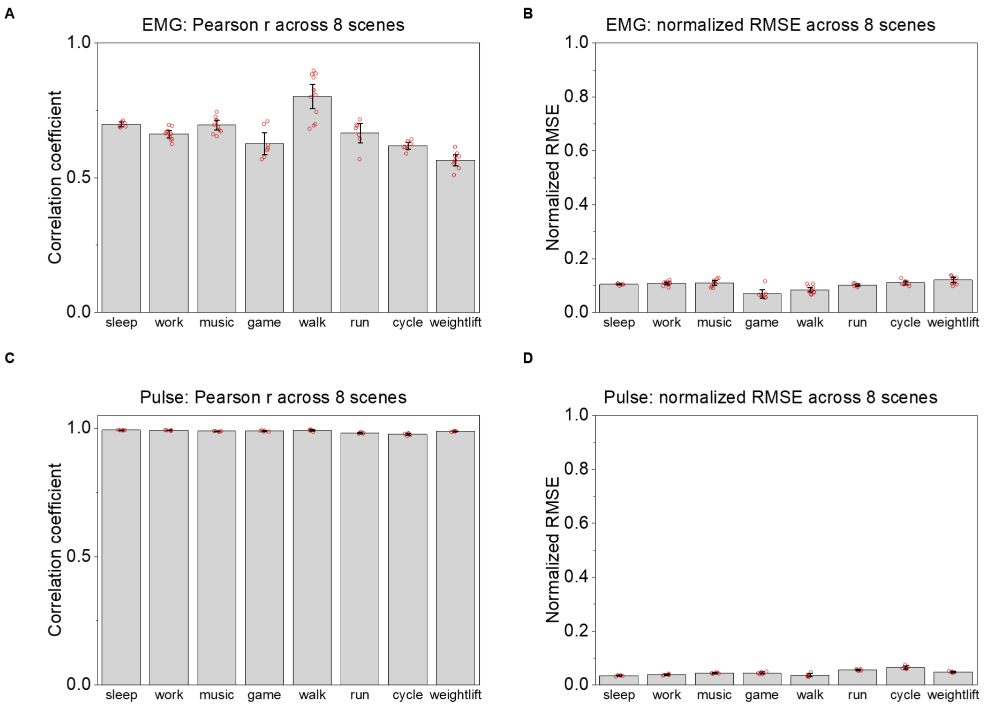

Fig. S6. Evaluation of representation learning framework to unseen subjects across diverse real-world scenarios. (**A** and **B**) Pearson correlation coefficient (A) and normalized root-mean-square error (RMSE) (B) between the original and reconstructed EMG signals across eight scenes (sleep, work, music, game, walk, run, cycle, and weightlifting). (**C** and **D**) Pearson correlation coefficient (C) and normalized RMSE (D) between the original and reconstructed peripheral pulse signals across the same eight scenes.

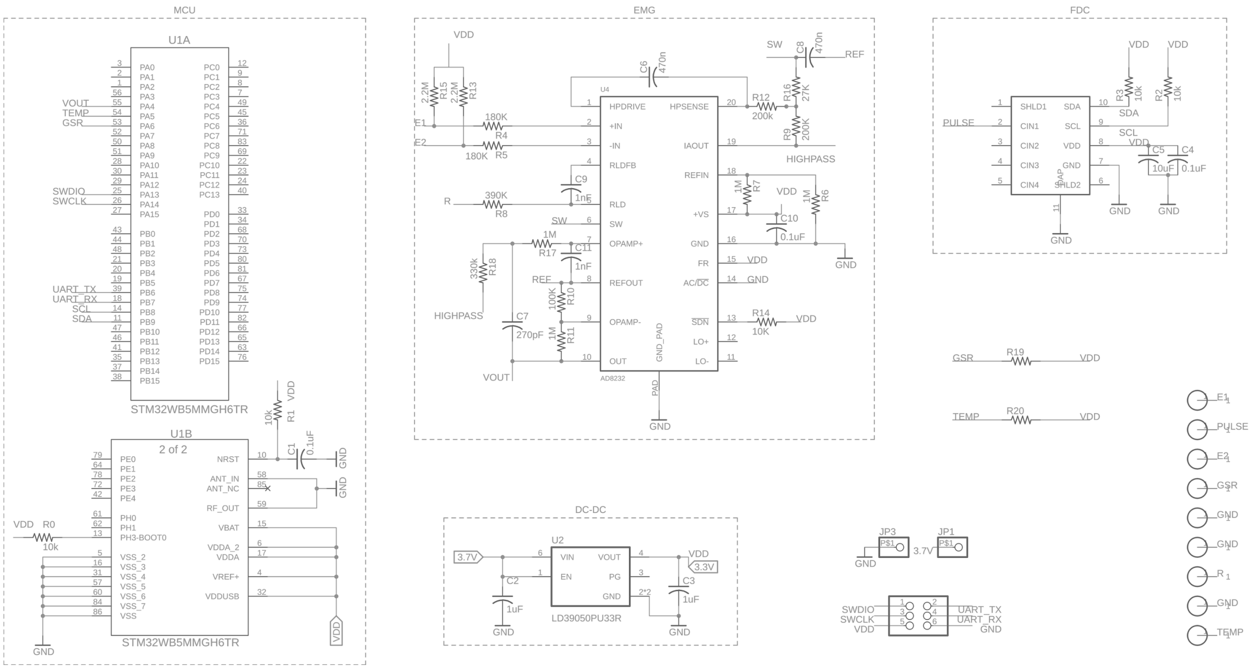

Fig. S7. Circuit schematic of ARISE electronic system.

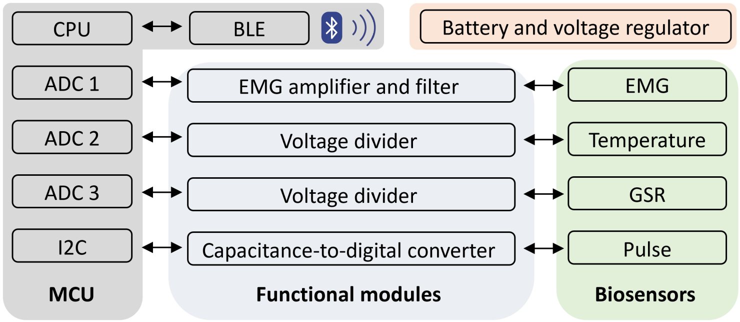

Fig. S8. Schematic diagram of the integrated electronic system. ADC, analog-to-digital converter; BLE, Bluetooth Low Energy.

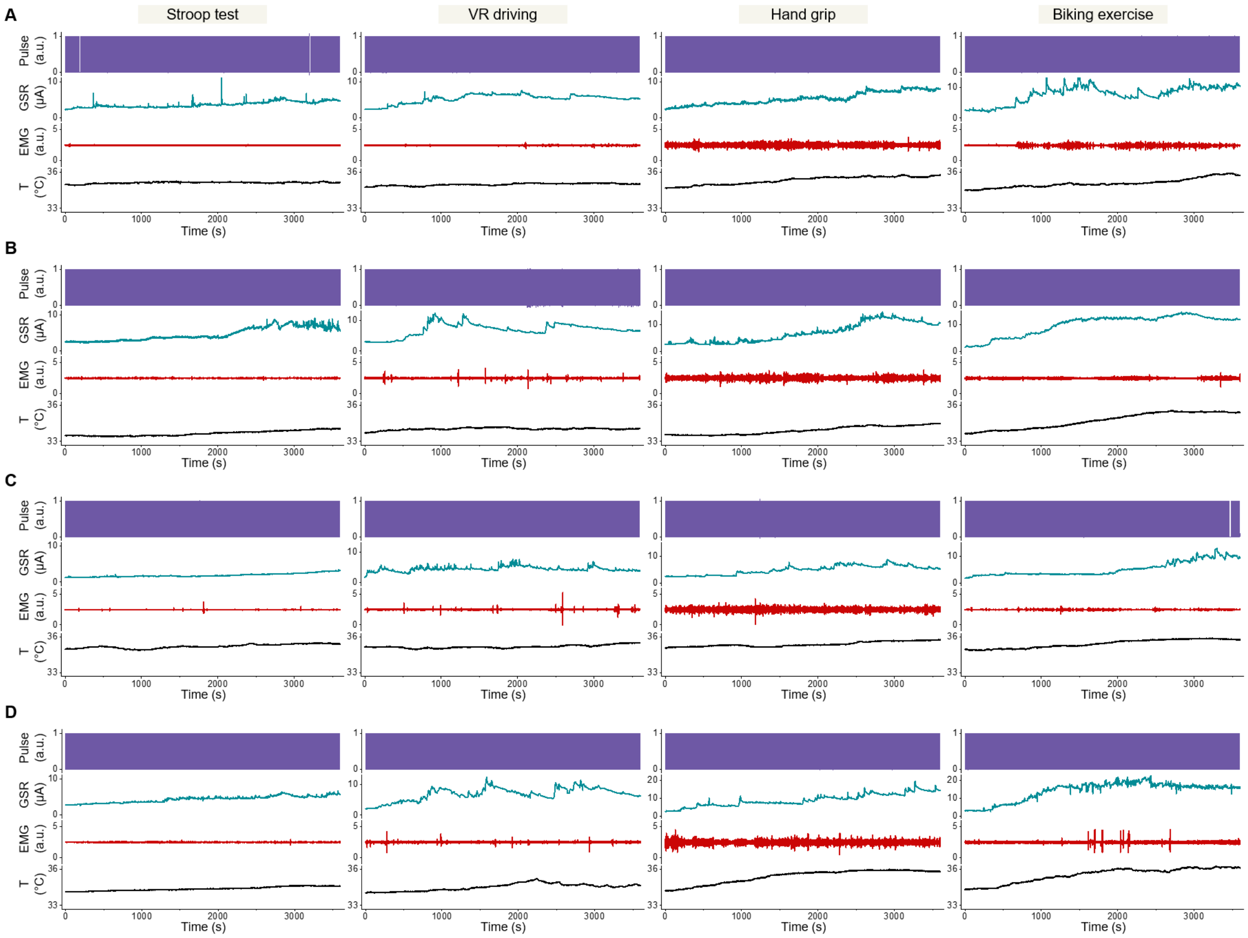

Fig. S9. Wearable data of controlled human studies.

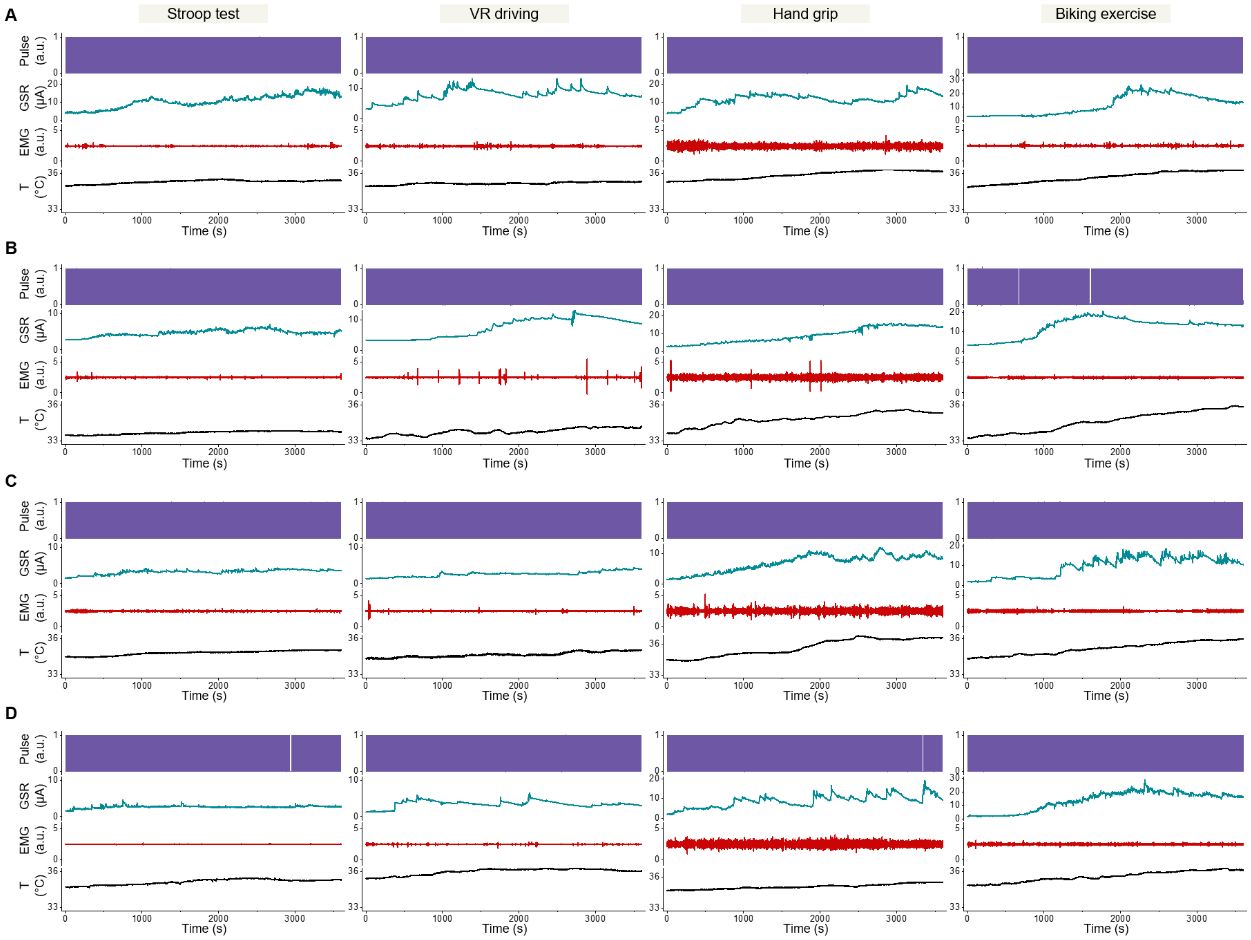

Fig. S10. Wearable data of controlled human studies (continued).

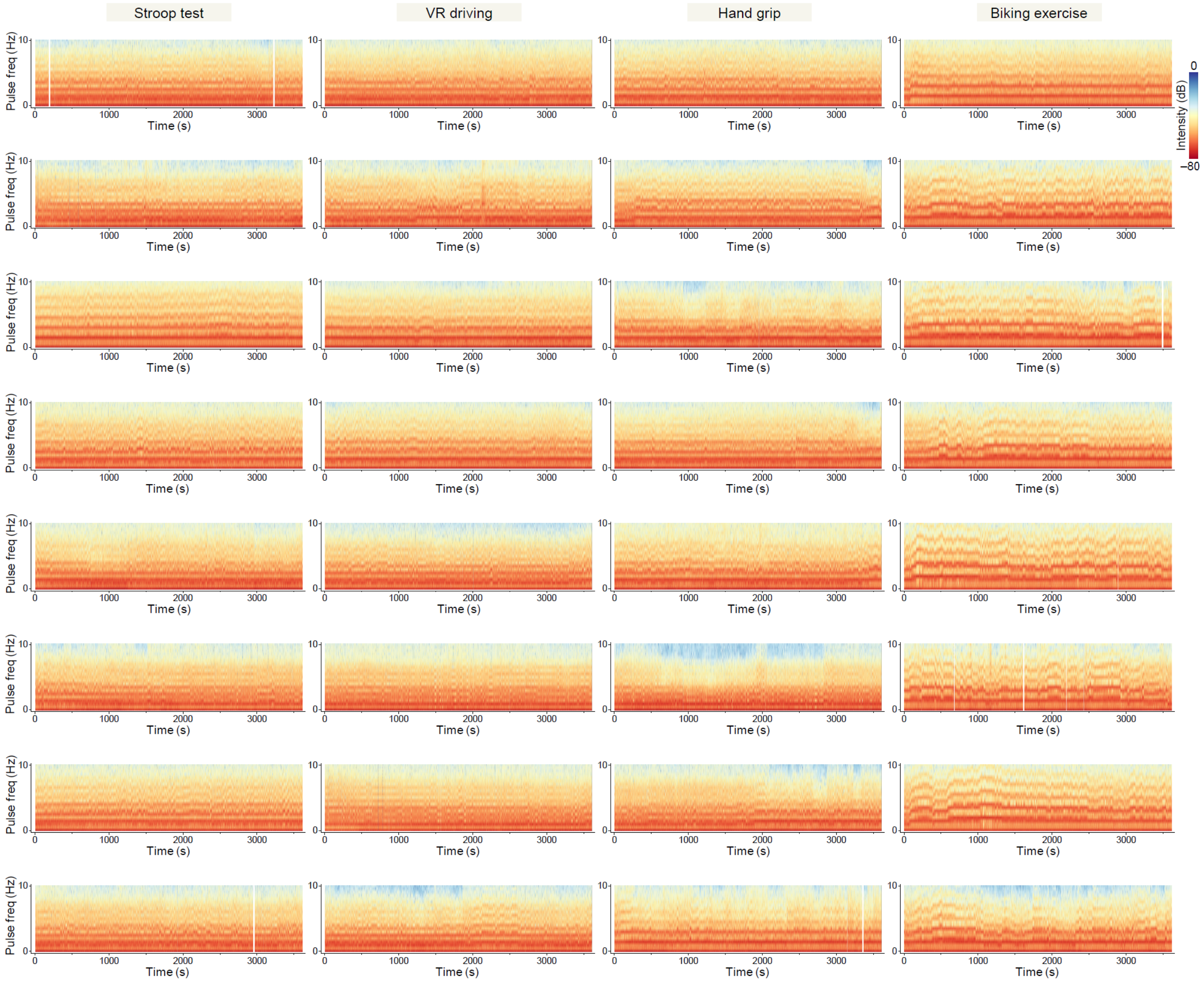

Fig. S11. Spectrogram of raw peripheral pulse data.

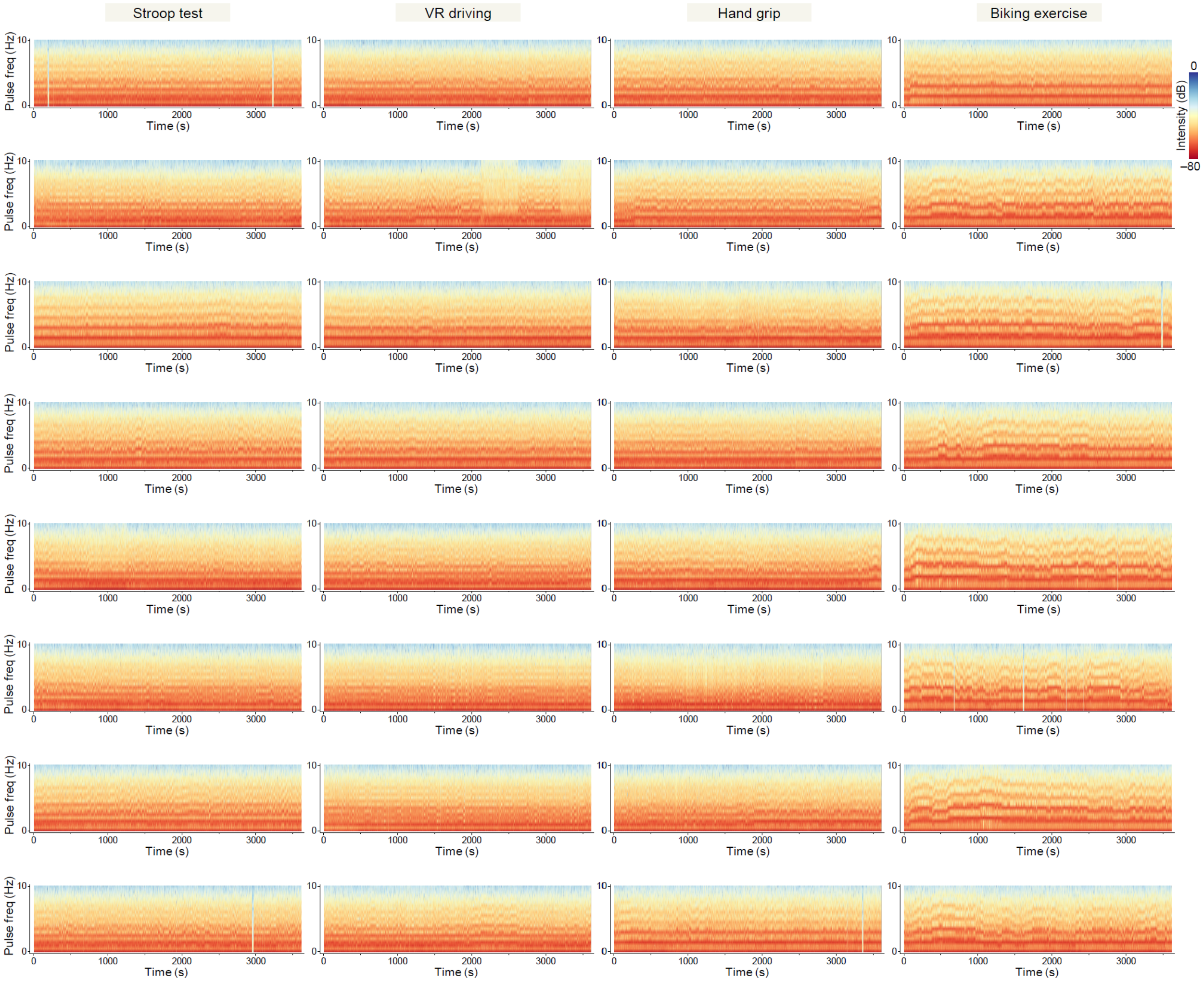

Fig. S12. Spectrogram of reconstructed pulse data with SVAE.

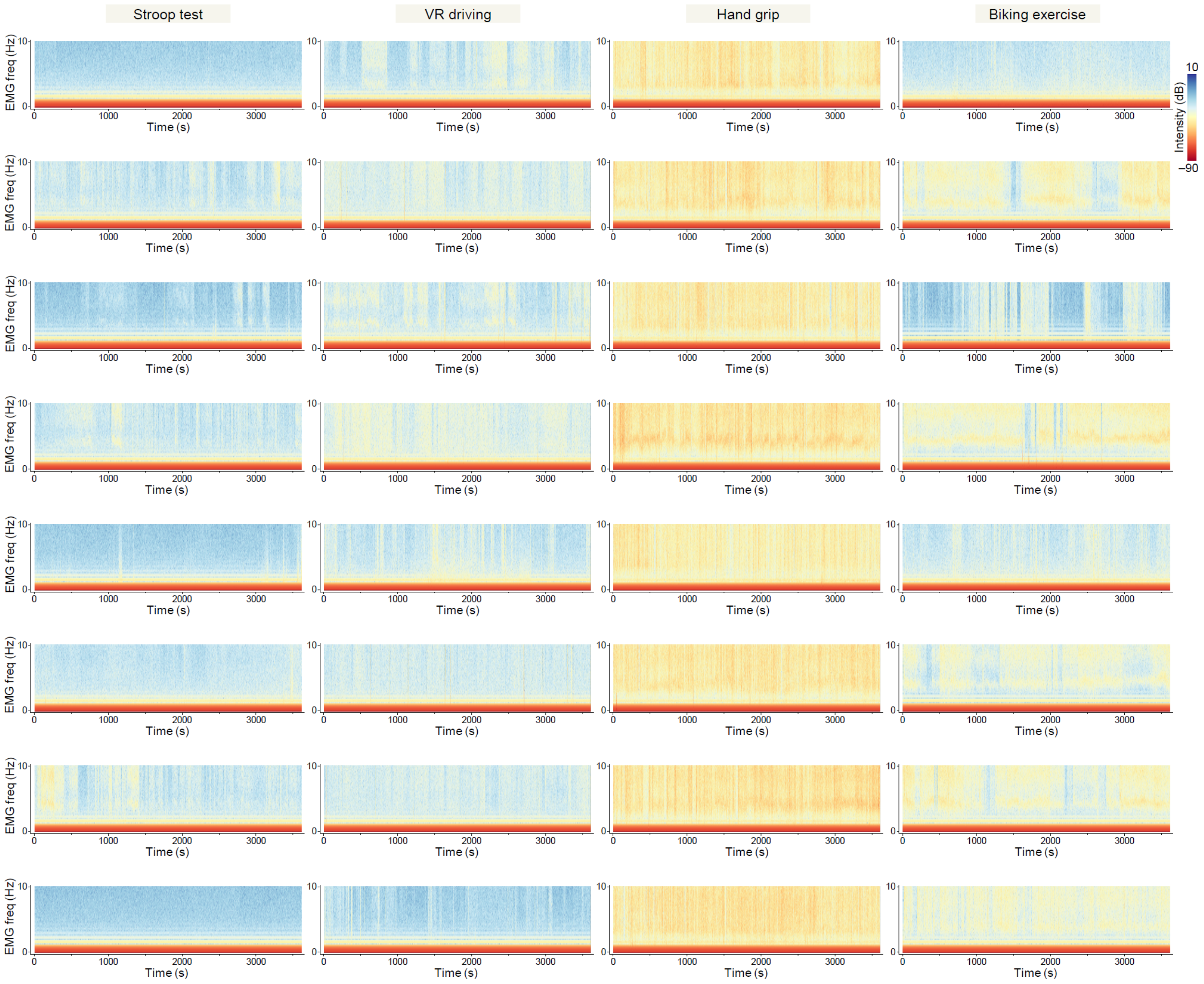

Fig. S13. Spectrogram of raw EMG data.

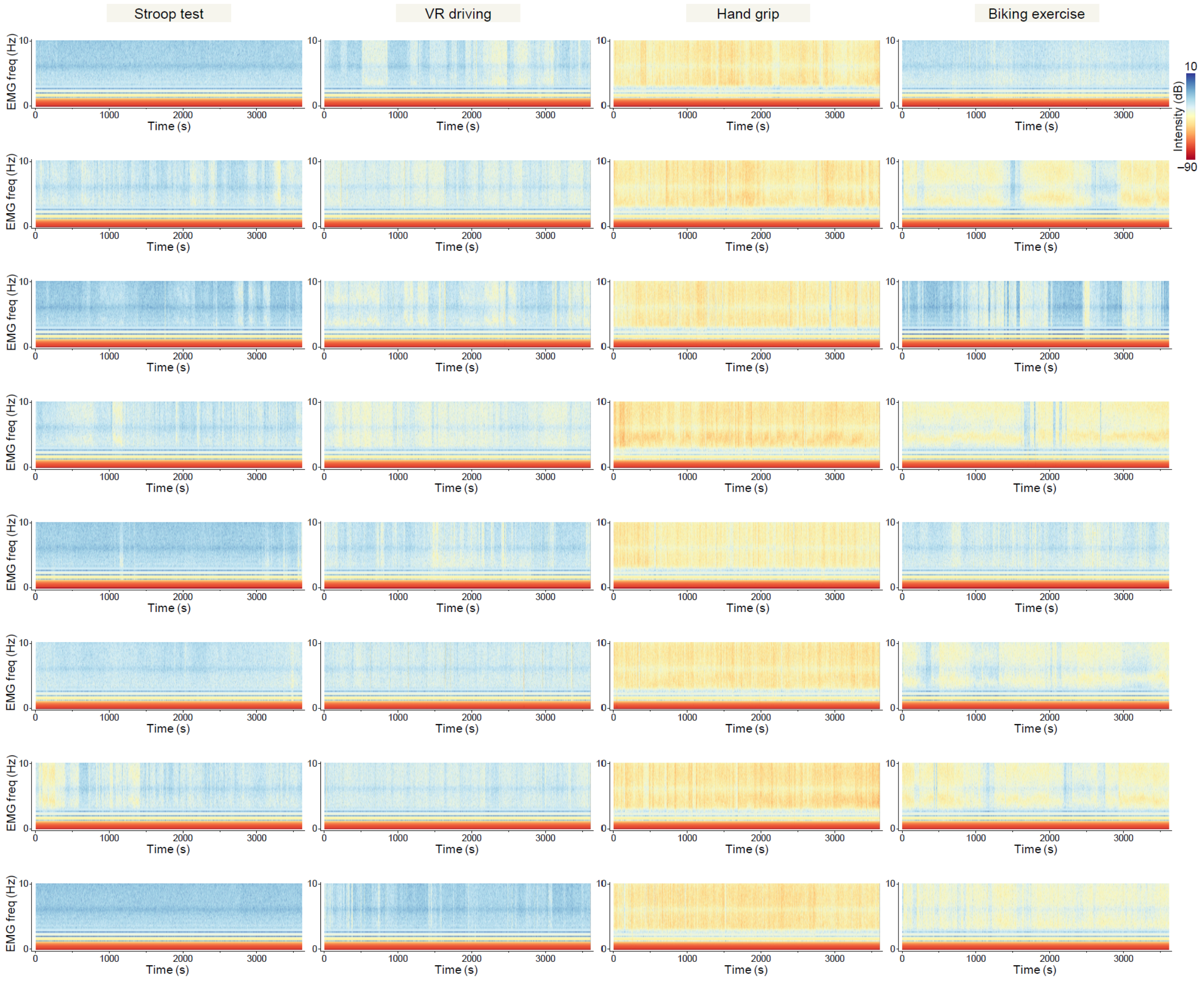

Fig. S14. Spectrogram of reconstructed EMG data with SVAE.

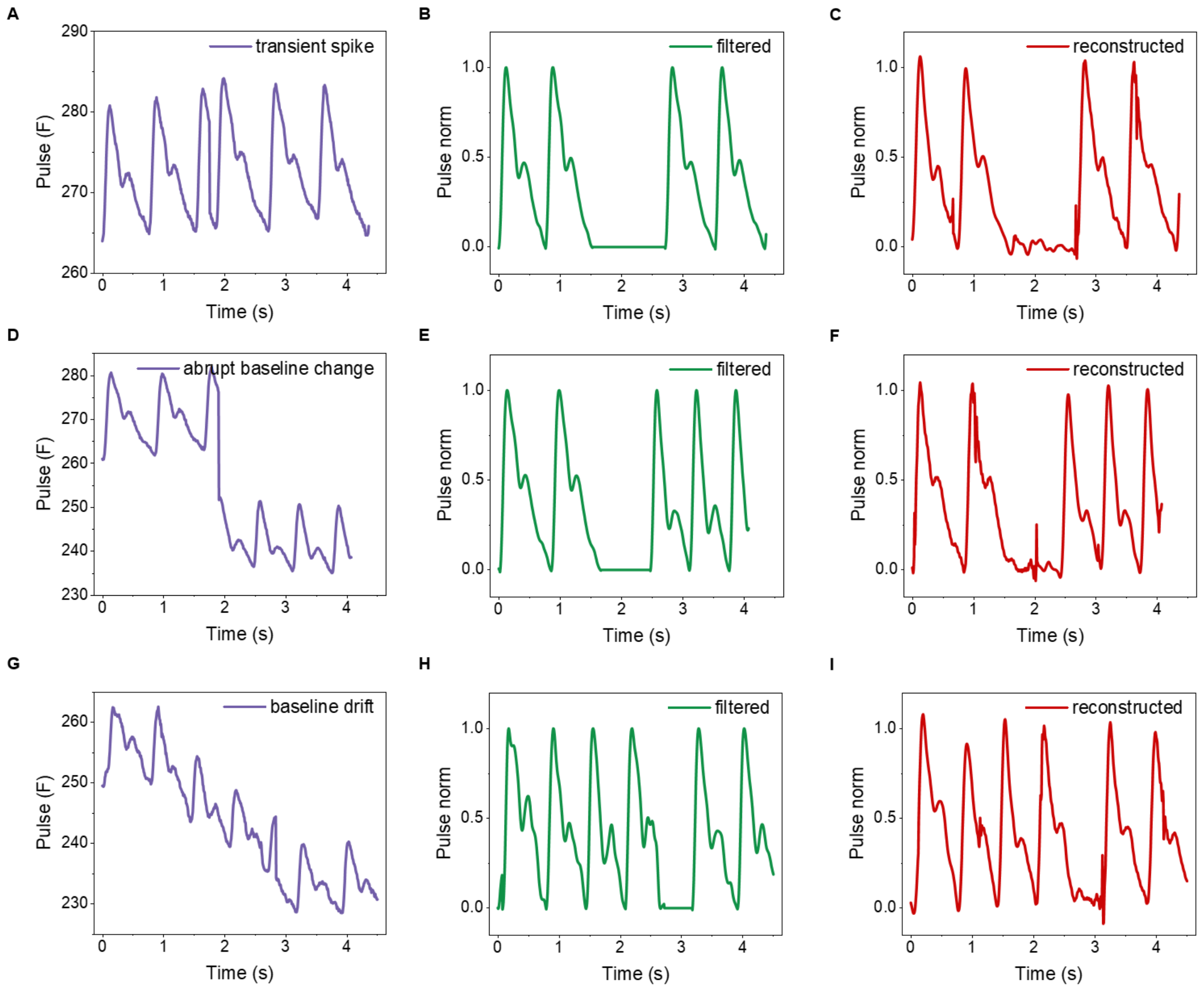

Fig. S15. Evaluation of pulse sensor stability and data pipeline robustness against representative motion artifacts during long-term wearing. (**A** to **C**) Pulse signals with a transient spike artifact, showing the raw corrupted signal (A), the filtered and normalized signal (B), and the SVAE-reconstructed signal (C). (**D** to **F**) Pulse signals with an abrupt baseline change, showing the raw corrupted signal (D), the filtered and normalized signal (E), and the SVAE-reconstructed signal (F). (**G** to **I**) Pulse signals with baseline drift, showing the raw corrupted signal (G), the filtered and normalized signal (H), and the SVAE-reconstructed signal (I).

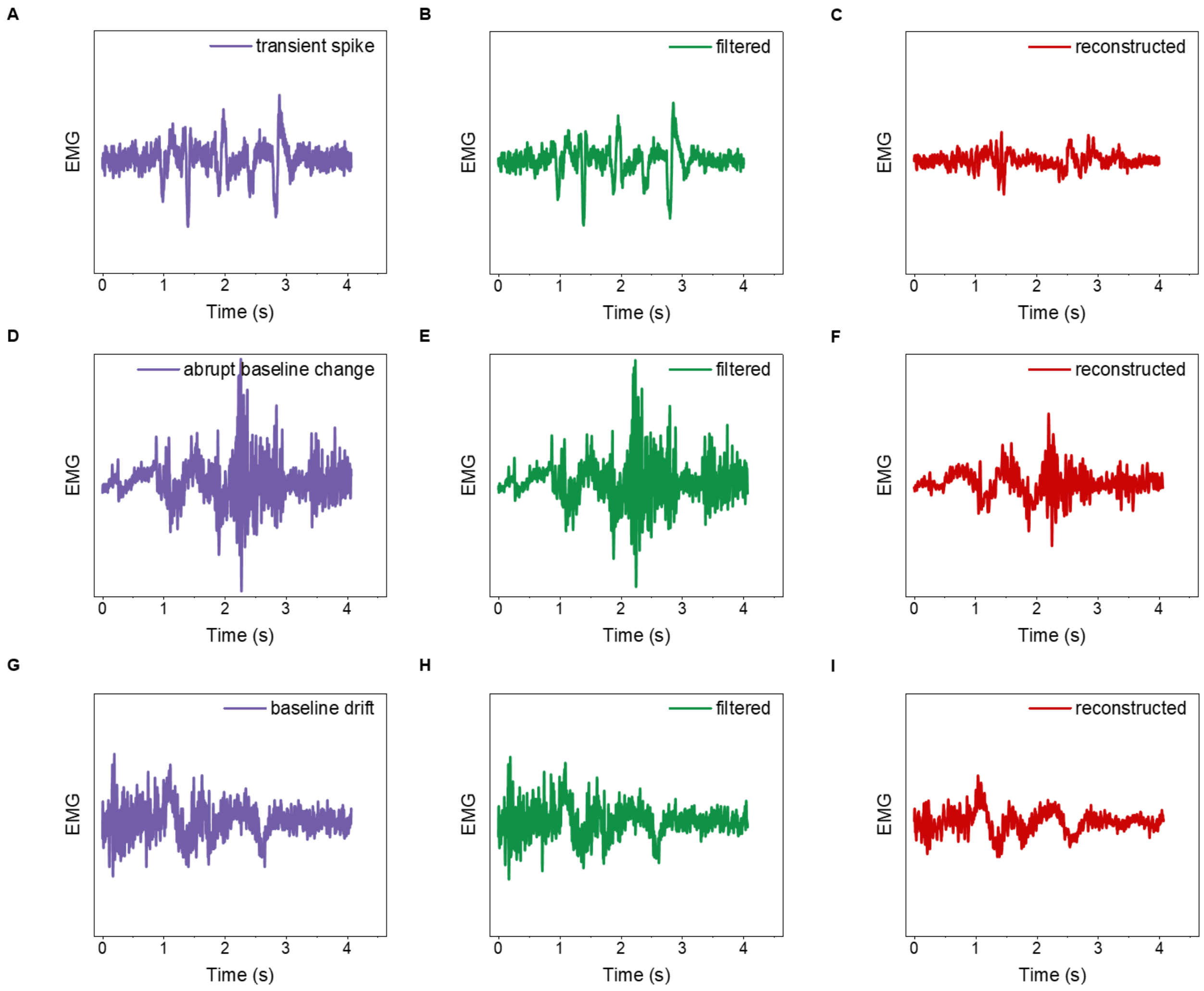

Fig. S16. Evaluation of EMG sensor stability and data-pipeline robustness against representative motion artifacts during long-term wear. (**A** to **C**) EMG signals with a transient spike artifact, showing the raw corrupted signal (A), the preprocessed signal after powerline-noise removal and segmentation (B), and the SVAE-reconstructed signal (C). (**D** to **F**) EMG signals with an abrupt baseline change, showing the raw corrupted signal (D), the preprocessed signal (E), and the SVAE-reconstructed signal (F). (**G** to **I**) EMG signals with baseline drift, showing the raw corrupted signal (G), the preprocessed signal (H), and the SVAE-reconstructed signal (I).

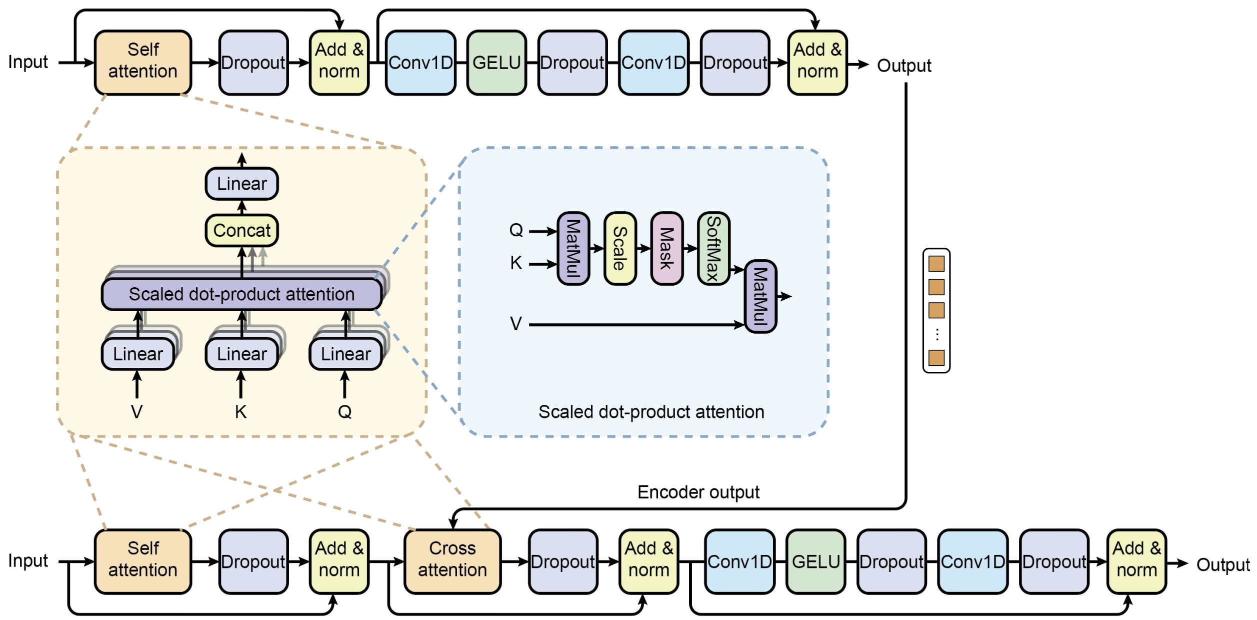

Fig. S17. Details of transformer architecture for downstream time-series tasks. Illustration of detailed layer structure of the encoder-decoder model. Embedded temporal signals are encoded with multi-head attention, followed by Conv1D, GELU and Dropout blocks.

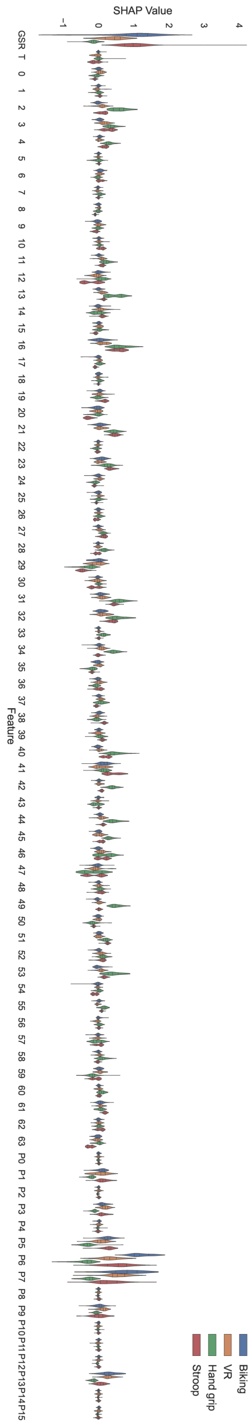

Fig. S18. Violin plot of SHAP values distribution across daily activities. GSR, galvanic skin response; T, temperature; features labeled from 0–63 corresponds to EMG sensors; features labeled from P0–P15 corresponds to peripheral pulse sensors.

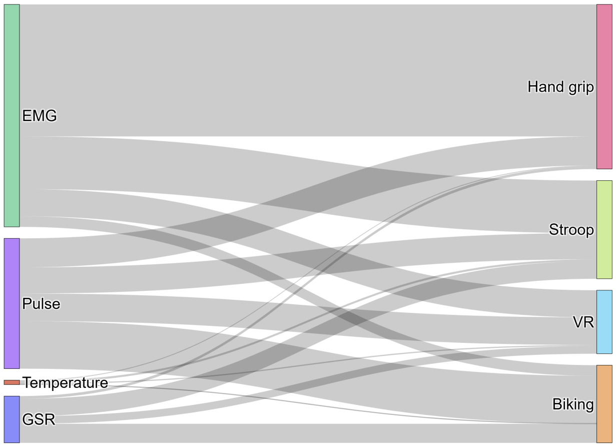

Fig. S19. SHAP summary plot of physiological sensors to daily activities.

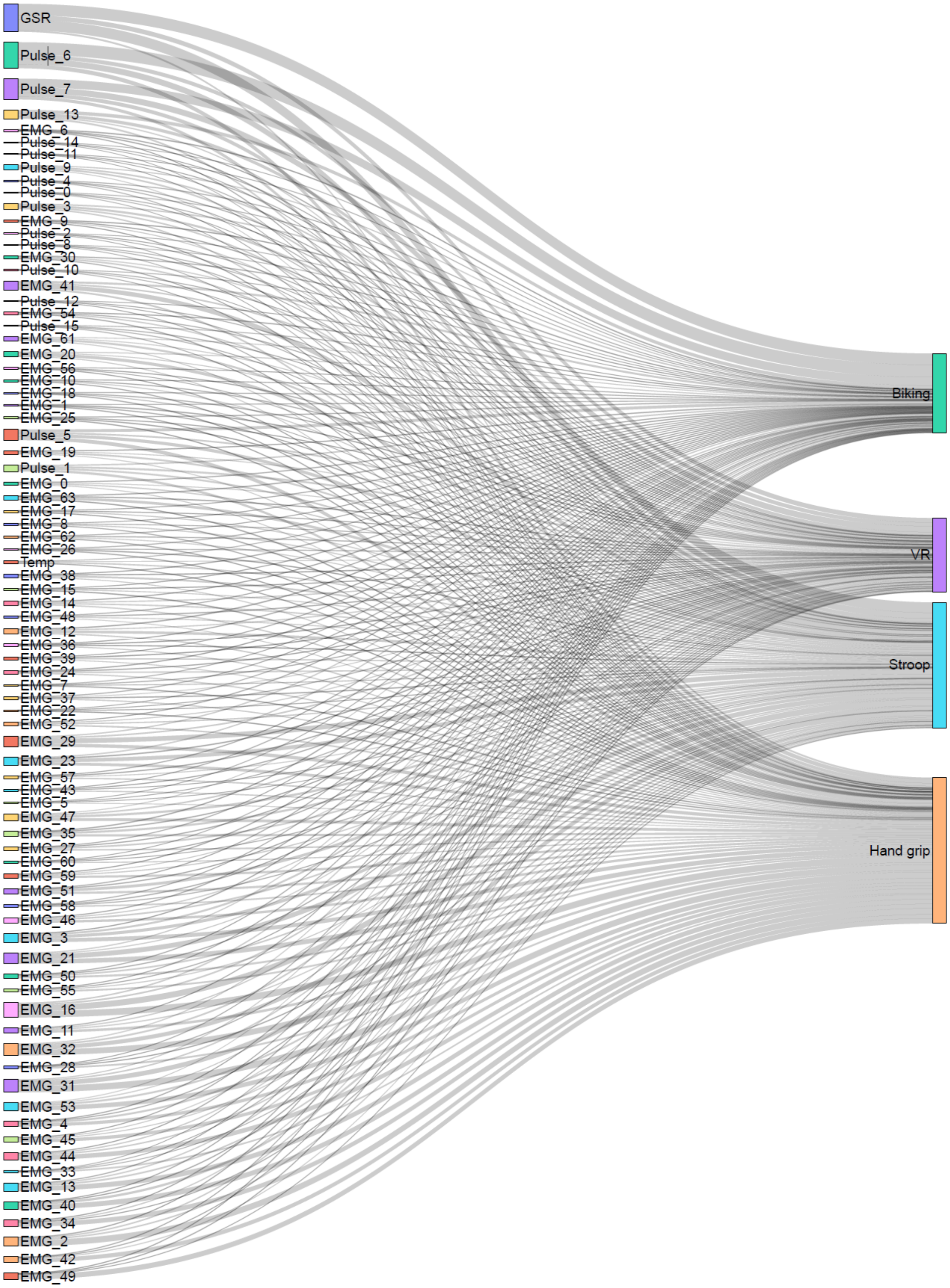

Fig. S20. Feature correlations of SVAE learnt representations with respect to activity class.

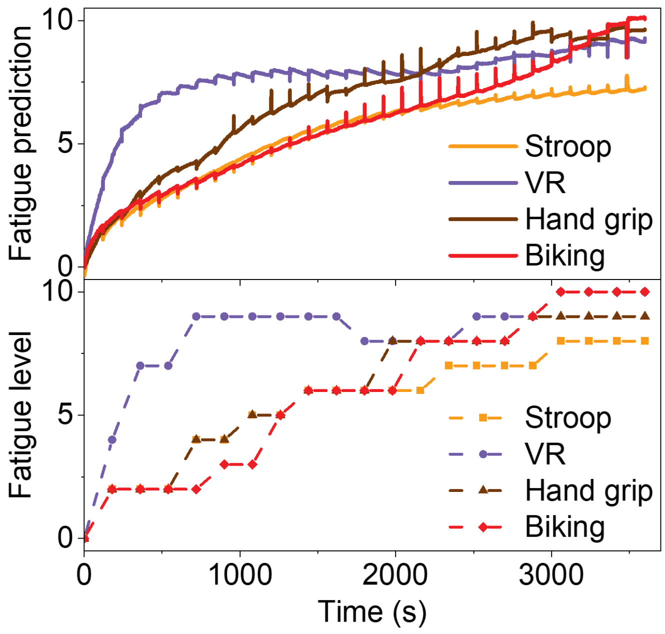

Fig. S21. Fatigue prediction based on time-series wearable data. Data from an unseen new subject.

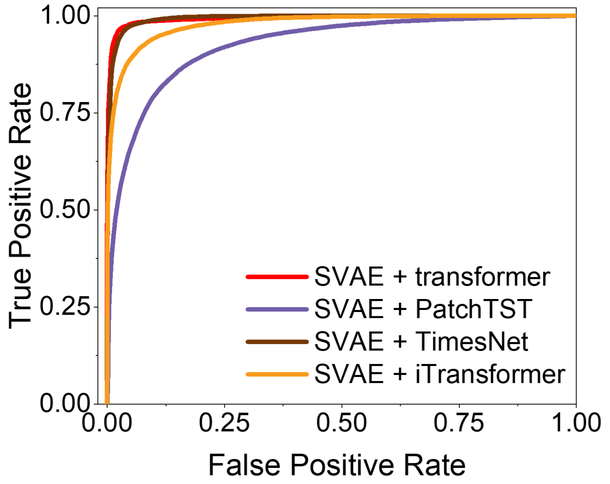

Fig. S22. Comparison of transformer with recent time-series architectures. ROC curve comparison of the proposed SVAE-transformer model with representative recent time-series models, including PatchTST, TimesNet, and iTransformer.

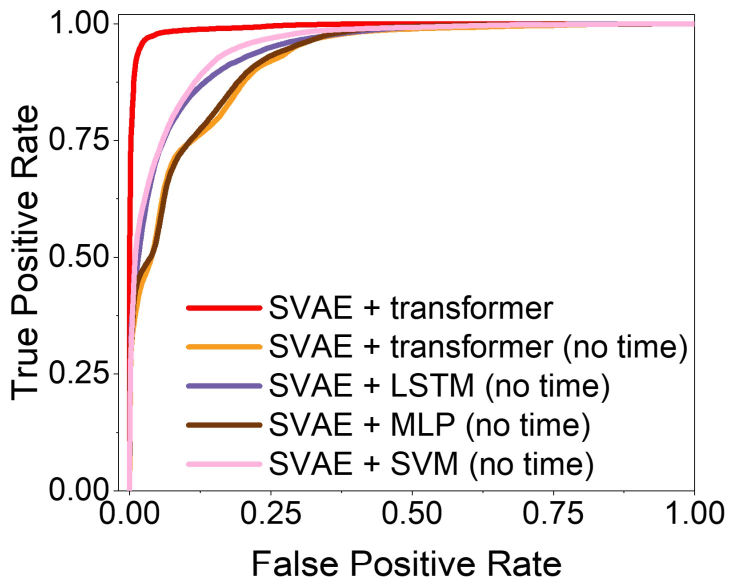

Fig. S23. ROC curve when temporal dependency is deprived in modeling.

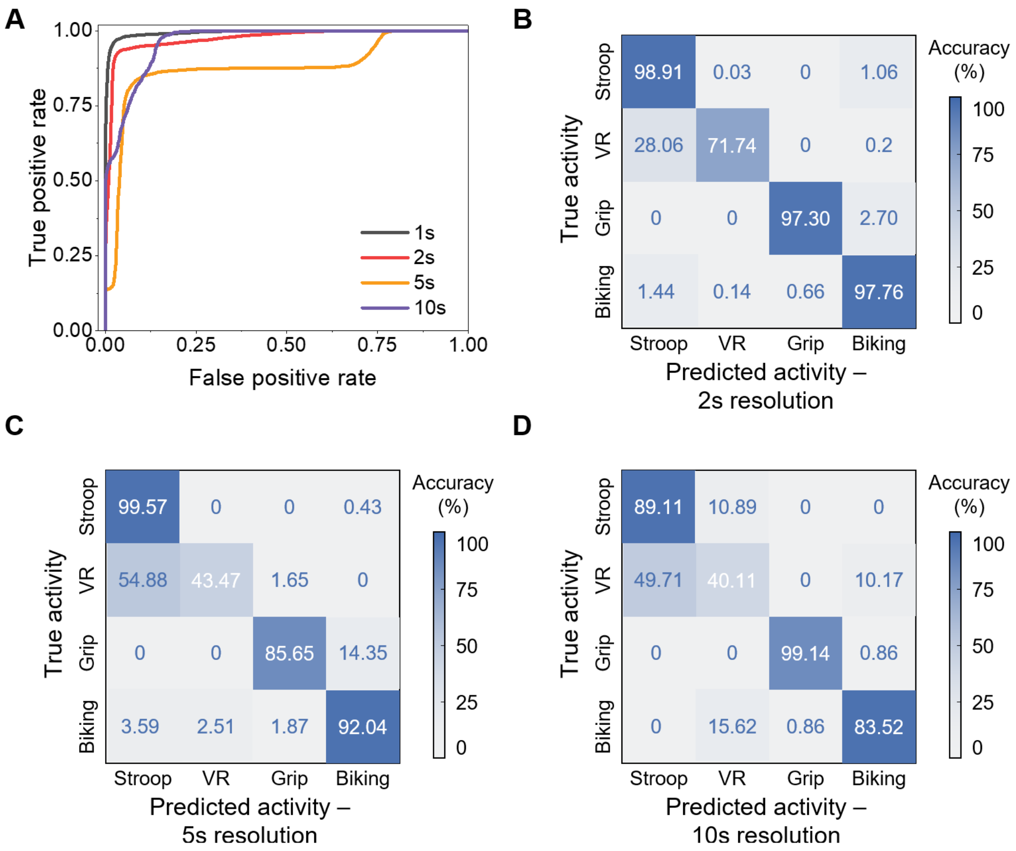

Fig. S24. Temporal-resolution sensitivity analysis of the SVAE-transformer model. (**A**) ROC curve comparison of activity recognition performance using SVAE-derived tokens at 1-s, 2-s, 5-s, and 10-s temporal resolutions. (**B** to **D**) Confusion matrices of the activity recognition model using 2-s (B), 5-s (C), and 10-s (D) token resolutions. The model maintained relatively high classification performance at 2-s resolution, whereas coarser 5-s and 10-s resolutions resulted in reduced performance.

Table S1. Representative wearable signal-processing and machine-learning approaches for activity recognition, fatigue assessment, and related human performance tasks.

Task	Sensors	Signal processing methods	Machine learning models	Performance	Year	References
Driver fatigue	EEG	Manual features, mainly fuzzy entropy	GBDT	94.0% for driver fatigue detection	2018	(*50*)
Mental fatigue	ECG, GSR	Manual features from ECG and GSR	DT	89% for fatigue prediction	2020	(*51*)
Running fatigue	Strain sensors	Manual features, such as MAV, min, max	RF	96% in participant-specific scenarios	2020	(*52*)
Hand gesture recognition	EMG	Manual features, such as MAV	Hyperdimensional computing	97.12% for 13 hand gesture recognition; 92.87% for 21 gesture recognition; 9.5% accuracy recovery after adaptive update	2021	(*44*)
Muscle fatigue	EMG	Manual features, mainly spectral correlation density coefficients	MLP+ELM	94% for binary classification	2022	(*53*)
Muscle fatigue	EMG	Manual features, such as MAV and RMS	N/A	N/A	2023	(*54*)
Degree of impairment regarding alcohol	Heart rate, T, alcohol	Manual features, such as HR, T	Ridge regression	86.6% ~ 90.2% depending on tasks	2023	(*55*)
Fatigue and human performance	IMU	Manual features, such as triaxial acceleration, angular velocity, and magnetic orientation	LSTM	59% for binary classification	2024	(*56*)
Fatigue detection	PPG	Manual features, such as HR, eyelid movement, and yawning frequency	CNN+LSTM	98.2% for binary classification	2024	(*57*)
Fatigue classification	PPG, blood cortisol	Manual features, mainly HRV and cortisol	NN (TabNet)	74.1% ~ 77.4% depending on feature selections	2025	(*58*)
Fatigue classification	Magnetoelastic sensor	Manual features of six eye-blink parameters	1D-CNN	96.4% for binary classification	2025	(*59*)
Fatigue assessment	ECG	Manual features, such as RR intervals	CNN+LSTM	83.3% for fatigue classification	2025	(*60*)
Fatigue assessment and human performance	Pulse, GSR, EMG, T	Representation learning	SVAE+transformer	94.7% for activity recognition; 90.2% for fatigue assessment		This work

EEG, electroencephalography. ECG, electrocardiography. EMG, electromyography. PPG, photoplethysmography. IMU, inertial measurement unit. MAV, mean absolute value. RMS, root mean square. HR, heart rate. HRV, heart rate variability. DT, decision tree. GBDT, gradient boosted decision tree. RF, random forest. MLP, multilayer perceptron. ELM, extreme learning machine. LSTM, long short-term memory. NN, neural network. CNN, convolutional neural network.

Table S2. Representative multimodal wearable hardware for activity recognition, fatigue assessment, and related human performance tasks.

Task	Sensors	Structure	Fabrication	Robustness	Sensing performance	Year	References
Tactile motions	Ionic receptor	Sensor arrays	Spin coating	12h sensing	Prone to motion artifacts	2020	(*61*)
Physiological body profiles	Blood pressure, chemical sensors	Multimodal patch + direct skin contact	Screen printing	1h sensing	Need to attach to neck, prone to movements	2021	(*62*)
Degree of impairment regarding alcohol	Heart rate, T, alcohol	Multimodal patch + 3 additional layers of microfluidics + energy storage layer	3D printing	12h sensing, not reusable	Prone to motion artifacts	2023	(*55*)
Stress response	Pulse, GSR, T, chemical sensors	Multimodal patch + 3 additional layers of microfluidics	Inkjet printing + laser cut	24h sensing, not reusable	Prone to motion artifacts	2024	(*7*)
Fatigue classification	PPG, blood cortisol	Medicore SA-3000P system + venous blood collection	N/A (commercial device)	N/A (commercial device)	Discrete data points	2025	(*58*)
Fatigue classification	Magneto elastic sensor	Single sensor + processing circuit layers	Spin coating	12h sensing	Need to attach on eyelids	2025	(*59*)
Sepsis diagnosis	Neuromorphic transistors	Multimodal patch + processing circuit layers	Inkjet printing	2h sensing	Discrete data points	2025	(*63*)
Fatigue assessment and human performance	Pulse, GSR, EMG, T	Multimodal patch + direct skin contact	Inkjet printing + laser cut	Multi-day sensing, reusable	Robust to motion artifacts		This work

Table S3. Comparisons between fatigue questionnaires and selection rationale.

Questionnaires	Outputs	Administration time	Validated populations	Validity	Consistency	References
Visual Analog Scale – Fatigue (VAS-F)	Fatigue, energy, sleepiness and mood states	< 1 minute for subscale	Healthy individuals and patients	Excellent	Excellent internal consistency	(*47*, *64–68*)
Chalder Fatigue Scale (CFQ)	Physical and mental fatigue	2 - 5 minutes	Adults 18-45 years, and patients with chronic fatigue syndrome	Good	Excellent internal consistency	(*69–71*)
Fatigue Severity Scale (FSS)	The impact of fatigue on functioning	2 - 3 minutes	Patients with medical and neurological disorders	Excellent	Excellent internal consistency	(*66*, *72*, *73*)
Multidimensional Assessment of Fatigue (MAF)	Four dimensions of fatigue: severity, distress, timing and interference	5 minutes	Adults with rheumatoid arthritis and other chronic diseases	Excellent	Excellent internal consistency	(*74–76*)
Short Form 36 (SF-36)	Fatigue, energy level, and effect on activities	< 1 minute for subscale	Healthy individuals and patients	Good	Excellent internal consistency	(*77*, *78*)

Movie Captions

Movie S1. Continuous and mobile wearable monitoring.

Movie S2. End-to-end time-series health evaluation.

REFERENCES

1. S. Sundaram, P. Kellnhofer, Y. Li, J.-Y. Zhu, A. Torralba, W. Matusik, Learning the signatures of the human grasp using a scalable tactile glove. *Nature* **569**, 698–702 (2019).

2. Y. Luo, Y. Li, P. Sharma, W. Shou, K. Wu, M. Foshey, B. Li, T. Palacios, A. Torralba, W. Matusik, Learning human–environment interactions using conformal tactile textiles. *Nat. Electron.* **4**, 193–201 (2021).

3. K. K. Kim, M. Kim, K. Pyun, J. Kim, J. Min, S. Koh, S. E. Root, J. Kim, B.-N. T. Nguyen, Y. Nishio, S. Han, J. Choi, C.-Y. Kim, J. B.-H. Tok, S. Jo, S. H. Ko, Z. Bao, A substrate-less nanomesh receptor with meta-learning for rapid hand task recognition. *Nat. Electron.* **6**, 64–75 (2023).

4. Q. Yang, W. Jin, Q. Zhang, Y. Wei, Z. Guo, X. Li, Y. Yang, Q. Luo, H. Tian, T.-L. Ren, Mixed-modality speech recognition and interaction using a wearable artificial throat. *Nat. Mach. Intell.* **5**, 169–180 (2023).

5. H. Hu, H. Huang, M. Li, X. Gao, L. Yin, R. Qi, R. S. Wu, X. Chen, Y. Ma, K. Shi, C. Li, T. M. Maus, B. Huang, C. Lu, M. Lin, S. Zhou, Z. Lou, Y. Gu, Y. Chen, Y. Lei, X. Wang, R. Wang, W. Yue, X. Yang, Y. Bian, J. Mu, G. Park, S. Xiang, S. Cai, P. W. Corey, J. Wang, S. Xu, A wearable cardiac ultrasound imager. *Nature* **613**, 667–675 (2023).

6. Y. Yang, Y. Yuan, G. Zhang, H. Wang, Y.-C. Chen, Y. Liu, C. G. Tarolli, D. Crepeau, J. Bukartyk, M. R. Junna, A. Videnovic, T. D. Ellis, M. C. Lipford, R. Dorsey, D. Katabi, Artificial intelligence-enabled detection and assessment of Parkinson’s disease using nocturnal breathing signals. *Nat. Med.* **28**, 2207–2215 (2022).

7. C. Xu, Y. Song, J. R. Sempionatto, S. A. Solomon, Y. Yu, H. Y. Y. Nyein, R. Y. Tay, J. Li, W. Heng, J. Min, A. Lao, T. K. Hsiai, J. A. Sumner, W. Gao, A physicochemical-sensing electronic skin for stress response monitoring. *Nat. Electron.* **7**, 168–179 (2024).

8. H. C. Ates, P. Q. Nguyen, L. Gonzalez-Macia, E. Morales-Narváez, F. Güder, J. J. Collins, C. Dincer, End-to-end design of wearable sensors. *Nat. Rev. Mater.* **7**, 887–907 (2022).

9. C. Xu, S. A. Solomon, W. Gao, Artificial intelligence-powered electronic skin. *Nat. Mach. Intell.* **5**, 1344–1355 (2023).

10. B. Zhang, J. Li, J. Zhou, L. Chow, G. Zhao, Y. Huang, Z. Ma, Q. Zhang, Y. Yang, C. K. Yiu, J. Li, F. Chun, X. Huang, Y. Gao, P. Wu, S. Jia, H. Li, D. Li, Y. Liu, K. Yao, R. Shi, Z. Chen, B. L. Khoo, W. Yang, F. Wang, Z. Zheng, Z. Wang, X. Yu, A three-dimensional liquid diode for soft, integrated permeable electronics. *Nature* **628**, 84–92 (2024).

11. H. Wang, T. Fu, Y. Du, W. Gao, K. Huang, Z. Liu, P. Chandak, S. Liu, P. Van Katwyk, A. Deac, A. Anandkumar, K. Bergen, C. P. Gomes, S. Ho, P. Kohli, J. Lasenby, J. Leskovec, T.-Y. Liu, A. Manrai, D. Marks, B. Ramsundar, L. Song, J. Sun, J. Tang, P. Veličković, M. Welling, L. Zhang, C. W. Coley, Y. Bengio, M. Zitnik, Scientific discovery in the age of artificial intelligence. *Nature* **620**, 47–60 (2023).

12. B. M. Lake, M. Baroni, Human-like systematic generalization through a meta-learning neural network. *Nature* **623**, 115–121 (2023).

13. S. Kumar, I. Dasgupta, N. D. Daw, J. D. Cohen, T. L. Griffiths, Disentangling abstraction from statistical pattern matching in human and machine learning. *PLOS Comput. Biol.* **19**, e1011316 (2023).

14. A. Vaswani, N. Shazeer, N. Parmar, J. Uszkoreit, L. Jones, A. N. Gomez, Ł. Kaiser, I. Polosukhin, Attention is all you need. *Adv. Neural Inf. Process. Syst.* **30**, 5998–6008 (2017).

15. T. Brown, B. Mann, N. Ryder, M. Subbiah, J. D. Kaplan, P. Dhariwal, A. Neelakantan, P. Shyam, G. Sastry, A. Askell, S. Agarwal, A. Herbert-Voss, G. Krueger, T. Henighan, R. Child, A. Ramesh, D. Ziegler, J. Wu, C. Winter, C. Hesse, M. Chen, E. Sigler, M. Litwin, S. Gray, B. Chess, J. Clark, C. Berner, S. McCandlish, A. Radford, I. Sutskever, D. Amodei, Language models are few-shot learners. *Adv. Neural Inf. Process. Syst.* **33**, 1877–1901 (2020).

16. T. Zhou, P. Niu, X. Wang, L. Sun, R. Jin, One fits all: Power general time series analysis by pretrained LM. *Adv. Neural Inf. Process. Syst.* **36**, 43322–43355 (2023).

17. H. Wu, T. Hu, Y. Liu, H. Zhou, J. Wang, M. Long, "TimesNet: Temporal 2D-variation modeling for general time series analysis," in *International Conference on Learning Representations* (ICLR, 2023).

18. P. Trirat, Y. Shin, J. Kang, Y. Nam, J. Na, M. Bae, J. Kim, B. Kim, J.-G. Lee, Universal time-series representation learning: A survey. arXiv:2401.03717 [cs.LG] (2024).

19. A. Das, W. Kong, R. Sen, Y. Zhou, A decoder-only foundation model for time-series forecasting. arXiv:2310.10688 [cs.LG] (2024).

20. A. Santoro, S. Bartunov, M. Botvinick, D. Wierstra, T. Lillicrap, "Meta-learning with memory-augmented neural networks," in *Proceedings of the 33rd International Conference on International Conference on Machine Learning* (JMLR.org, 2016).

21. S. Ambike, F. Paclet, V. M. Zatsiorsky, M. L. Latash, Factors affecting grip force: Anatomy, mechanics, and referent configurations. *Exp. Brain Res.* **232**, 1219–1231 (2014).

22. D. P. Kingma, M. Welling, "Auto-encoding variational Bayes," in *2nd International Conference on Learning Representations* (ICLR, 2014).

23. R. H. C. E. Souza, E. L. M. Naves, Attention detection in virtual environments using EEG signals: A scoping review. *Front. Physiol.* **12**, 727840 (2021).

24. A. Ioannou, E. Papastavrou, M. N. Avraamides, A. Charalambous, Virtual reality and symptoms management of anxiety, depression, fatigue, and pain: A systematic review. *SAGE Open Nurs.* **6**, 2377960820936163 (2020).

25. E. Chang, H. T. Kim, B. Yoo, Virtual reality sickness: A review of causes and measurements. *Int. J. Hum. Comput. Interact.* **36**, 1658–1682 (2020).

26. R. M. Broxterman, J. C. Craig, J. R. Smith, S. L. Wilcox, C. Jia, S. Warren, T. J. Barstow, Influence of blood flow occlusion on the development of peripheral and central fatigue during small muscle mass handgrip exercise. *J. Physiol.* **593**, 4043–4054 (2015).

27. T. C. Barbosa, A. C. Machado, I. D. Braz, I. A. Fernandes, L. C. Vianna, A. C. L. Nobrega, B. M. Silva, Remote ischemic preconditioning delays fatigue development during handgrip exercise. *Scand. J. Med. Sci. Sports* **25**, 356–364 (2015).

28. S. Day, *Important Factors in Surface EMG Measurement* (Bortec Biomedical Ltd., 2002).

29. M. B. I. Reaz, M. S. Hussain, F. Mohd-Yasin, Techniques of EMG signal analysis: Detection, processing, classification and applications. *Biol. Proced. Online* **8**, 11–35 (2006).

30. M. Behrens, M. Gube, H. Chaabene, O. Prieske, A. Zenon, K.-C. Broscheid, L. Schega, F. Husmann, M. Weippert, Fatigue and human performance: An updated framework. *Sports Med.* **53**, 7–31 (2023).

31. S. A. Sharples, J. A. Gould, M. S. Vandenberk, J. M. Kalmar, Cortical mechanisms of central fatigue and sense of effort. *PLOS ONE* **11**, e0149026 (2016).

32. R. C. Coetzer, G. P. Hancke, "Driver fatigue detection: A survey," in *AFRICON 2009* (IEEE, 2009), pp. 1–6.

33. S. M. Marcora, W. Staiano, V. Manning, Mental fatigue impairs physical performance in humans. *J. Appl. Physiol.* **106**, 857–864 (2009).

34. S. C. Segerstrom, L. S. Nes, Heart rate variability reflects self-regulatory strength, effort, and fatigue. *Psychol. Sci.* **18**, 275–281 (2007).

35. L. Nybo, P. Rasmussen, Inadequate cerebral oxygen delivery and central fatigue during strenuous exercise. *Exerc. Sport Sci. Rev.* **35**, 110–118 (2007).

36. J. González-Alonso, C. Teller, S. L. Andersen, F. B. Jensen, T. Hyldig, B. Nielsen, Influence of body temperature on the development of fatigue during prolonged exercise in the heat. *J. Appl. Physiol.* **86**, 1032–1039 (1999).

37. G. Sjøgaard, G. Savard, C. Juel, Muscle blood flow during isometric activity and its relation to muscle fatigue. *Eur. J. Appl. Physiol.* **57**, 327–335 (1988).

38. M. Körber, A. Cingel, M. Zimmermann, K. Bengler, Vigilance decrement and passive fatigue caused by monotony in automated driving. *Procedia Manuf.* **3**, 2403–2409 (2015).

39. R. Hooda, V. Joshi, M. Shah, A comprehensive review of approaches to detect fatigue using machine learning techniques. *Chronic Dis. Transl. Med.* **8**, 26–35 (2022).

40. H. Zhang, M. Cisse, Y. N. Dauphin, D. Lopez-Paz, "mixup: Beyond empirical risk minimization," in *6th International Conference on Learning Representations* (ICLR, 2018).

41. H. Yao, Y. Wang, L. Zhang, J. Y. Zou, C. Finn, C-Mixup: Improving generalization in regression. *Adv. Neural Inf. Process. Syst.* **35**, 3361–3376 (2022).

42. S. Talukder, Y. Yue, G. Gkioxari, TOTEM: TOkenized Time Series EMbeddings for general time series analysis. arXiv:2402.16412 [cs.LG] (2024).

43. A. Das, W. Kong, A. Leach, S. K. Mathur, R. Sen, R. Yu, "Long-term forecasting with TiDE: Time-series dense encoder," in *Transactions on Machine Learning Research* (TMLR, 2023).

44. A. Moin, A. Zhou, A. Rahimi, A. Menon, S. Benatti, G. Alexandrov, S. Tamakloe, J. Ting, N. Yamamoto, Y. Khan, F. Burghardt, L. Benini, A. C. Arias, J. M. Rabaey, A wearable biosensing system with in-sensor adaptive machine learning for hand gesture recognition. *Nat. Electron.* **4**, 54–63 (2021).

45. Y. Nie, N. H. Nguyen, P. Sinthong, J. Kalagnanam, "A time series is worth 64 words: Long-term forecasting with transformers," in *International Conference on Learning Representations* (ICLR, 2023).

46. Y. Liu, T. Hu, H. Zhang, H. Wu, S. Wang, L. Ma, M. Long, "iTransformer: Inverted transformers are effective for time series forecasting," in *International Conference on Learning Representations* (ICLR, 2024).

47. K. A. Lee, G. Hicks, G. Nino-Murcia, Validity and reliability of a scale to assess fatigue. *Psychiatry Res.* **36**, 291–298 (1991).

48. D. Khanna, J. E. Pope, P. P. Khanna, M. Maloney, N. Samedi, D. Norrie, G. Ouimet, R. D. Hays, The minimally important difference for the fatigue visual analog scale in patients with rheumatoid arthritis followed in an academic clinical practice. *J. Rheumatol.* **35**, 2339–2343 (2008).

49. D. P. Kingma, J. Ba, “Adam: A method for stochastic optimization,” in *3rd International Conference on Learning Representations* (ICLR, 2015).

50. J. Hu, J. Min, Automated detection of driver fatigue based on EEG signals using gradient boosting decision tree model. *Cogn. Neurodyn.* **12**, 431–440 (2018).

51. Z. Zeng, Z. Huang, K. Leng, W. Han, H. Niu, Y. Yu, Q. Ling, J. Liu, Z. Wu, J. Zang, Nonintrusive monitoring of mental fatigue status using epidermal electronic systems and machine-learning algorithms. *ACS Sens.* **5**, 1305–1313 (2020).

52. M. Gholami, C. Napier, A. G. Patiño, T. J. Cuthbert, C. Menon, Fatigue monitoring in running using flexible textile wearable sensors. *Sensors* **20**, 5573 (2020).

53. K. Divya Bharathi, P. A. Karthick, S. Ramakrishnan, Automated detection of muscle fatigue conditions from cyclostationary based geometric features of surface electromyography signals. *Comput. Methods Biomech. Biomed. Eng.* **25**, 320–332 (2022).

54. Q. Gong, X. Jiang, Y. Liu, M. Yu, Y. Hu, A flexible wireless sEMG system for wearable muscle strength and fatigue monitoring in real time. *Adv. Electron. Mater.* **9**, 2200916 (2023).

55. Y. Song, R. Y. Tay, J. Li, C. Xu, J. Min, E. Shirzaei Sani, G. Kim, W. Heng, I. Kim, W. Gao, 3D-printed epifluidic electronic skin for machine learning–powered multimodal health surveillance. *Sci. Adv.* **9**, eadi6492 (2023).

56. A. Biró, A. I. Cuesta-Vargas, L. Szilágyi, AI-assisted fatigue and stamina control for performance sports on IMU-generated multivariate times series datasets. *Sensors* **24**, 132 (2024).

57. L. Kong, K. Xie, K. Niu, J. He, W. Zhang, Remote photoplethysmography and motion tracking convolutional neural network with bidirectional long short-term memory: Non-invasive fatigue detection method based on multi-modal fusion. *Sensors* **24**, 455 (2024).

58. J. E. Kim, N. H. Kim, S. K. Choi, J.-Y. Lee, K. Lee, J. S. Han, Machine learning-based fatigue classification using heart rate variability and cortisol: A multimodal approach to wearable health monitoring. *Digit. Health* **11**, 20552076251395570 (2025).

59. J. Xu, C. Duan, X. Wan, Z. Che, X. Zhao, Y. Zhou, Y. Song, J. Yin, T. Tat, S. Li, J. Chen, A soft magnetoelastic sensor to decode levels of fatigue. *Nat. Electron.* **8**, 709–720 (2025).

60. Y. Wang, R. Dang, B. Hu, Q. Wang, Deep learning-based fatigue monitoring in natural environments: Multi-level fatigue state classification. *Bioengineering* **12**, 1374 (2025).

61. I. You, D. G. Mackanic, N. Matsuhisa, J. Kang, J. Kwon, L. Beker, J. Mun, W. Suh, T. Y. Kim, J. B.-H. Tok, Z. Bao, U. Jeong, Artificial multimodal receptors based on ion relaxation dynamics. *Science* **370**, 961–965 (2020).

62. J. R. Sempionatto, M. Lin, L. Yin, E. De la Paz, K. Pei, T. Sonsa-Ard, A. N de Loyola Silva, A. A. Khorshed, F. Zhang, N. Tostado, S. Xu, J. Wang, An epidermal patch for the simultaneous monitoring of haemodynamic and metabolic biomarkers. *Nat. Biomed. Eng.* **5**, 737–748 (2021).

63. Y. Choi, P. Jin, S. Lee, Y. Song, R. Y. Tay, G. Kim, J. Yoo, H. Han, J. Yeom, J. H. Cho, D.-H. Kim, W. Gao, All-printed chip-less wearable neuromorphic system for multimodal physicochemical health monitoring. *Nat. Commun.* **16**, 5689 (2025).

64. M. A. García-Pérez, R. Alcalá-Quintana, Accuracy and precision of responses to visual analog scales: Inter- and intra-individual variability. *Behav. Res.* **55**, 4369–4381 (2023).

65. B. Y. Tseng, B. J. Gajewski, P. M. Kluding, Reliability, responsiveness, and validity of the visual analog fatigue scale to measure exertion fatigue in people with chronic stroke: A preliminary study. *Stroke Res. Treat.* **2010**, e412964 (2010).

66. S.-R. Shin, A.-L. Han, Improved chronic fatigue symptoms after removal of mercury in patient with increased mercury concentration in hair toxic mineral assay: A case. *Korean J. Fam. Med.* **33**, 320–325 (2012).

67. A. M. Abbasi, M. Motamedzade, M. Aliabadi, R. Golmohammadi, L. Tapak, Study of the physiological and mental health effects caused by exposure to low-frequency noise in a simulated control room. *Build. Acoust.* **25**, 233–248 (2018).

68. S. Lee, M. Kim, H. Jung, D. Kwon, S. Choi, H. You, Effects of a motion seat system on driver's passive task-related fatigue: An on-road driving study. *Sensors* **20**, 2688 (2020).

69. T. Chalder, G. Berelowitz, T. Pawlikowska, L. Watts, S. Wessely, D. Wright, E. P. Wallace, Development of a fatigue scale. *J. Psychosom. Res.* **37**, 147–153 (1993).

70. G. B. Neuberger, Measures of fatigue: The fatigue questionnaire, fatigue severity scale, multidimensional assessment of fatigue scale, and short form-36 vitality (energy/fatigue) subscale of the short form health survey. *Arthritis Care Res.* **49**, S175–S183 (2003).

71. A. Deale, K. Husain, T. Chalder, S. Wessely, Long-term outcome of cognitive behavior therapy versus relaxation therapy for chronic fatigue syndrome: A 5-year follow-up study. *Am. J. Psychiatry* **158**, 2038–2042 (2001).

72. L. B. Krupp, N. G. LaRocca, J. Muir-Nash, A. D. Steinberg, The fatigue severity scale. Application to patients with multiple sclerosis and systemic lupus erythematosus. *Arch. Neurol.* **46**, 1121–1123 (1989).

73. J. E. Schwartz, L. Jandorf, L. B. Krupp, The measurement of fatigue: A new instrument. *J. Psychosom. Res.* **37**, 753–762 (1993).

74. B. B. Tack, "Dimensions and correlates of fatigue in older adults with rheumatoid arthritis," thesis, UCSF (1991).

75. B. L. Belza, C. J. Henke, E. H. Yelin, W. V. Epstein, C. L. Gilliss, Correlates of fatigue in older adults with rheumatoid arthritis. *Nurs. Res.* **42**, 93–99 (1993).

76. J. Bormann, M. Shively, T. L. Smith, A. L. Gifford, Measurement of fatigue in HIV-positive adults: Reliability and validity of the global fatigue index. *J. Assoc. Nurses AIDS Care* **12**, 75–83 (2001).

77. J. E. Ware, C. D. Sherbourne, The MOS 36-item short-form health survey (SF-36). I. Conceptual framework and item selection. *Med. Care* **30**, 473–483 (1992).

78. B. Gandek, J. E. Ware, N. K. Aaronson, J. Alonso, G. Apolone, J. Bjorner, J. Brazier, M. Bullinger, S. Fukuhara, S. Kaasa, A. Leplège, M. Sullivan, Tests of data quality, scaling assumptions, and reliability of the SF-36 in eleven countries: Results from the IQOLA Project. *J. Clin. Epidemiol.* **51**, 1149–1158 (1998).
